# Navigating the ethical landscape of scholarly publishing: a comparative evaluation of Gemini and DeepSeek LLMs in addressing authorship and contributorship disputes

**DOI:** 10.3389/frma.2026.1781697

**Published:** 2026-04-08

**Authors:** Kannan Sridharan, Gowri Sivaramakrishnan

**Affiliations:** 1 Department of Pharmacology and Therapeutics, College of Medicine and Health Sciences, Arabian Gulf University, Manama, Bahrain; 2 Bahrain Defence Force Royal Medical Services, Riffa, Bahrain

**Keywords:** authorship and contributorship, COPE guidelines, large language models, prompt engineering, publication ethics

## Abstract

**Background:**

The rising complexity of publication ethics, particularly authorship disputes, necessitates exploring Large Language Models (LLMs) as potential evaluative tools. This study compares the performance of Google Gemini 2.5 Flash and DeepSeek-V3.2 against expert Committee on Publication Ethics (COPE) forum responses.

**Methods:**

A cross-sectional analysis including 12 COPE authorship and contributorship cases was conducted using three prompting strategies: Minimal, Deterministic, and Stochastic. Responses were scored across seven domains on a 5-point Likert scale (1 = poor, 5 = excellent) by independent raters.

**Results:**

Both LLMs achieved perfect scores (5 ± 0) in Actionability of Recommendations and high marks in Safety and Avoidance of Hallucination (4.88 ± 0.33). In the Consistency with COPE Principles domain, DeepSeek performed slightly better than Gemini (4.45 ± 1.0 vs. 4.12 ± 1.29), while Gemini showed a better Overall Appropriateness (4.03 ± 0.98 vs. 3.82 ± 1.29) but they were not statistically significant. Both models struggled most with Identification of Ethical Issues (Gemini: 3.91 ± 1.33; DeepSeek: 3.82 ± 1.29). Under Minimal prompts, Gemini's ethical identification was lower (3.55 ± 1.44) compared to Deterministic/Stochastic prompts (4.09 ± 1.3). Qualitatively, Gemini recorded an 8% major disagreement rate with COPE, while DeepSeek had a 16% combined (minor and major) disagreement rate. Mean similarity scores to COPE forum experts were approximately 4 for both models. Both models missed specific legal/copyright nuances but provided unique “value-add” strategies, such as author disassociation statements and editorial de-escalation training, not present in original COPE forum advice.

**Conclusion:**

LLMs demonstrated high degree of alignment with COPE expert ethical reasoning. While they possess a “legal blind spot,” their ability to provide actionable and clear guidance, optimized through structured prompting, makes them valuable supplementary tools for journal editors.

## Introduction

Large language models (LLMs), developed based on natural language processing and deep learning methods, have revolutionized several streams including the healthcare sector in terms of clinical decision-making, developing patient education materials, medical education, and research (Alkalbani et al., 2025; Maity and Saikia, 2025). Beyond their utility in clinical data synthesis, these models are increasingly being scrutinized for their potential to navigate the complex ethical landscapes that define modern scholarly activity. A recent study that assessed LLMs across diverse case studies revealed that they can identify good clinical practice (GCP) issues, offer guidance on GCP violations, detect conflicts of interest and standard operating procedures deficiencies, recognize vulnerable populations, and suggest expedited review criteria (Sridharan and Sivaramakrishnan, 2024). Another study further established this capability, revealing that LLMs could potentially identify issues related to the suitability of proposed eligibility criteria for study participants, vulnerability issues, essential information to be disclosed in informed consent documents, risk–benefit assessments, and the justification of the use of a placebo in simulated high-risk medical research proposals (Sridharan and Sivaramakrishnan, 2025).

As the volume of global research output continues to escalate, the demand for standardized ethical oversight and expert guidance has never been greater. The Committee on Publication Ethics (COPE), established in 1997 with the aim of promoting integrity in scholarly research and its publication, provides an essential opportunity for journal editors to discuss and resolve complex ethical issues related to the publication process (Wager, 2012). Currently, COPE encompasses 14,500 members from various disciplines across 97 countries, serving as a global authority on ethical standards (COPE, COPE). COPE acts as a premier advisory body providing advice on publication ethics and promoting good editorial practices amongst journal editors and publishers. A cornerstone of COPE's influence is its dedicated forum, where members bring their specific cases related to publication ethics for thorough discussion and receive collective advice from the COPE Council and the broader membership (Foo and Wilson, 2012). To support this mission, COPE issues comprehensive guidance documents across essential topics, including journal management, research ethics, authorship and contributorship, data, plagiarism, peer review, and conflicts of interest. To date, 16 guidelines have been published, and seven guidelines have been endorsed by COPE, providing a robust framework for assisting journal editors and researchers alike (COPE Guidance, COPE Guidance). These resources serve a wide range of stakeholders, offering researchers a roadmap for ethical publishing, and providing readers, authors, and reviewers with the detailed information necessary to identify high-integrity journals (Lane, 2018).

Despite the presence of these established frameworks, authorship and contributorship disputes remain among the most intricate challenges in scholarly publishing, often involving nuanced human dynamics and varying interpretations of technical contributions. Given the burgeoning potential of LLMs to serve as virtual editorial assistants, it is imperative to rigorously evaluate whether these models can emulate the high-level reasoning provided by expert committees. Therefore, we conducted the present study to systematically compare the performance of LLMs against expert human benchmarks in real-world ethical scenarios. The primary objective of this study was to compare the quality, ethical alignment, and usefulness of responses produced by two prominent LLMs, Google Gemini and DeepSeek, against the consensus advice provided by COPE members for a set of anonymized editorial cases specifically related to authorship and contributorship issues. The secondary objectives were to evaluate the inter-model variability to determine consistency between different AI architectures and to assess the intra-model stability by examining how performance fluctuates across three distinct prompting variations: minimal, deterministic, and stochastic. Through this analysis, we sought to determine the reliability of LLMs in providing editorial guidance and the extent to which prompt engineering can optimize their alignment with established publication ethics standards.

## Methods

### Study design and ethical considerations

This study employed an observational, cross-sectional design to evaluate the performance of LLM against a set of anonymized case texts from the Committee on Publication Ethics (COPE). The methodological framework of this study involved evaluative research and comparative case study methodologies, that are commonly used in technology assessment, applied ethics, and human-computer interaction, like benchmarking a new diagnostic tool against expert judgment. The study protocol was prospectively registered in the Open Science Framework (Protocol for LLMs Assessing COPE Queries, Protocol for LLMs Assessing COPE Queries), and all evaluative procedures were conducted between November and December 2025. This research adhered to the latest Declaration of Helsinki guidelines; however, given that the study involved no interaction with human subjects and utilized publicly available COPE cases that are free from copyright restrictions, formal ethics committee approval was not required.

### Study procedure and case selection

The cases listed in the COPE forum were accessed in the present study (COPE Cases, COPE Cases). Cases were included if they met the following criteria:

*Inclusion criteria:*

- Cases listed between January 2020 until December 31, 2020.
- Cases listed in the domain on authorship and contributorship as filtered by COPE.
- Cases with a clear question/dilemma and at least one COPE member response.

*Exclusion criteria:*

- Cases with missing text or where COPE response is primarily administrative (e.g., “we will refer to our policies”) with no substantive guidance.

Two authors independently assessed the listed cases posted in COPE. For each case, the following details were obtained: original anonymized case description and COPE member response/s.

### Description of included cases

A total of 12 cases posted by the COPE members were as follows:

1. Author admits failure to credit other authors (Case 20-07)
2. Author displays bullying behavior toward handling editor (Case 20-08)
3. Authors requesting withdrawal of articles from similarity check database in order to re-publish (Case 20-10)
4. Unable to contact authors (Case 20-11)
5. Paper published without permission or acknowledgment from institution (Case 20-15)
6. Professional misconduct of one author (Case 20-17)
7. Conflict between two authors (Case 20-18)
8. Potentially fake academic affiliation (Case 20-20)
9. Data source for study of questionable integrity and provenance (Case 20-23)
10. Request for addition of new authors (Case 20-25)
11. Authorship order in dual publications (Case 20-29)
12. Conflicting authorship in a collaboration (Case 20-30)

Summaries of the cases including the COPE member response are outlined in Table 1.

**Table 1:** Key issues identified with authorship and contributorship domain in the included COPE cases.

Case number	Key issue	Key COPE forum responses	Outcome
20-07	• Article submitted by solo author accepted following peer review. • Post-publication the author wants to add more authors, and disagreement exists between the order of authors, particularly lead author. • Author wants to retract and resubmit with a new order of authors.	• Articles should not be retracted solely on the purpose of authorship issues. • If the data validity is not a concern, retraction should not be done. • Copyright infringement/legal issues to be clarified.	Republished with corrected authors list and a corrigendum.
20-08	• Manuscript rejected by the handling editor in consultation with the senior editor of a journal. • The corresponding author appealed stating that he has carried out a poll on social media on the relevance of the current manuscript to the journal compared to a previous paper published in the same journal, along with the request to provide detailed response for rejection based on a list of questions. • The manuscript was rejected again, following which the corresponding author again contacted the journal with expression of disappointment and threatened with freedom of information between the editors that led to the decision of rejection. • The Editors considered this as borderline bullying and informed senior author of the manuscript, who assured to act internally.	• State in the instructions to authors about the appeal process, and that the editorial deliberations are confidential and any formal enquiries to be made through a lawyer. • The author may also have difficult personality with misdirected enthusiasm who is engaged and respond to directions and education from the journal.	Not applicable.
20-10	• Manuscript was accepted in a paid open-access journal indexed in the Scopus database as warranted by the author's institution. • Following publication, the journal has been dropped from being indexed in the Scopus database. • The author requests retraction of the article from the journal and Crossref^®^ (from similarity check database).	• Retraction for non-ethical reasons is not in line with COPE guidelines. • Authors should admit and disclose this information in their next submission. • Negotiate with the institution stating that at the time of publication, the journal was indexed with Scopus database. • Crossref^®^ should not remove the article for this reason.	The case has been shared with Crossref^®^ and Turnitin^®^. A blog about the case will be posted soon and it will appear in the member newsletter.
20-11	• The corresponding author, co-authors and their institution were not responding to the Editorial queries post-acceptance of a manuscript.	• Provide a deadline for the author and the institution following which the article should be withdrawn. • Send a registered post to the author regarding the same. • Authors need to formally withdraw the article if they wish to submit it to another journal. • Legal and copyright violations shall exist if the author has not signed journal publishing agreement.	The journal has contacted all authors and their institutions providing a deadline beyond which the manuscript will be withdrawn. Considering the absence of any response, the journal withdrew the manuscript.
20-15	• The author affiliated with a research institution published a paper as a single author without acknowledging the affiliated institution. • The publisher was contacted by the research integrity officer with a letter of concern stating that a formal investigation revealed failure of the author to acknowledge fully the likely contributions made by other staff and students in his research group. • The investigation panel agreed unanimously that the author had behaved unprofessionally, and research misconduct had taken place. • The research institute further elaborated the inability to reach an agreement with the author and asked the publisher to publish an erratum. • The author initially did not agree to publishing an erratum but eventually requested modification in the erratum which was not acceptable to the author's institution.	• The institution should resolve this issue with the author. • Editor cannot adjudicate this issue. • Advised publishing an expression of concern until consensus is reached. • Editor may contact the second institution acknowledged by the author. • To threaten the author and the institution of retracting the article in case no agreement can be reached.	The author agreed to publish an erratum in a way that satisfied both the institutions.
20-17	• The co-author of a published article wishes to be removed due to allegations on the lead and corresponding author. • Due to violations in the code of conduct, the lead author of the article was banned by the organization and the coauthor wanted to remove his name due to this alleged behavior. • The ethical issue is to remove a coauthor from the article who has contributed and met the criteria for being an author in that article.	• The reason cited by the coauthor does not involve any of the science underlying in the article and so is not a matter of publication or research ethics. • According to the ICMJE criteria, removal of author should be based on academic reasons. • Coauthor to be encouraged to remain in the article. • If the complainant coauthor proves that the behavior of the lead author has affected the research with the backing up of the institution, then it can be escalated to scientific misconduct and possibly retraction.	Work on the guidance for cases of author behavioral misconduct is being drafted by the COPE.
20-18	• Following a publication of an article, a complaint is received by the editor that the individual did significant statistical analysis but was not included in the published article. • No written agreement but only verbal agreement according to the complaining author. • Despite several queries and phone calls to the editorial office and editor, the lead author maintains the stand that the complaining author did not contribute significantly to warrant authorship.	• Journals should not get involved in authorship disputes. • The author's institutions should be involved in resolving the dispute. • The editor shall inform the lead author about the COPE flowchart on addition of author following publication and COPE discussion document on authorship.	Not mentioned
20-20	• A journal (journal A) that published two articles from a group of authors was contacted by the editor of another journal (B) who rejected manuscripts from the same author group who stated receiving harassing emails from the same author. • Further, the editor of journal B stated that the specified address was erroneous and belonged to not-for-profit academic institution, and the provided telephone number did not work. • Following this email, the editor of Journal A rejected another manuscript of the same author group considering the legitimacy of the affiliation and the data.	• It is not correct for providing unsolicited information to a journal editor. • Suggests adherence to the COPE guidance on sharing information among editors-in-chief regarding possible misconduct. • It recommends the disclosing EIC to include a statement that “the information provided does not indicate a judgment of wrongdoing but is merely intended to alert EIC in case they have other information that might assist the handling of this case.” • Articles can be published by individuals from non-academic institutions, and the editor can independently assess the veracity of the data. • If the veracity of the data is questionable, the editor reserves the right to reject the paper.	Not mentioned
20-23	• A journal received an article related to COVID-19 pandemic and had favorable peer review reports.	• The editor should not hold the review process and is disingenuous to the authors.	Not mentioned
	• Just before the final discussion, it came to the journal's attention that the integrity and provenance of the data (on which this study is based) as well as the other published studies were based had expression of concern issued by journal editors. But there was no final verdict issued on the veracity of data source. • The editor has a dilemma of whether to reject the paper citing the expression of concern by the other source or should hold it so that it will not get published elsewhere.	• Possibly, the editor can reject the manuscript with strong statement that it should not be submitted elsewhere until the data source has been vetted. • Another option is to communicate to the author to wait until the investigation is completed and inform the editor on the results of the investigation, following which a decision can be rendered. • The journal can also fetch the data and seek advice from the experts on the veracity. • Possibility of false declaration at the submission time should also be explored.	
20-25	• An article by two authors underwent peer review following which it was accepted, copyediting completed and the proof was shared with the authors. • A request was received from the corresponding author for adding two authors who assisted during the revision of the manuscript as the author suffered an injury.	• Authors can be added during the proofreading stage provided appropriate justification is provided and agreed by all authors. • Authors must submit proof that justifies that the additional author has met all the authorship criteria specified in ICMJE. • If they do not meet the ICMJE criteria, COPE suggests acknowledgment.	Not mentioned
20-29	• Expert groups from two societies produced a guideline consensus that must be published separately in the journals of each society with different authorship orders.	• Consensus publications should be identical and contain the same order of authors in all simultaneous publications. • Alternatively, the authorship can be given as a collective group with the individuals being listed separately in variant order across journals. • If joint publication with the same order is not acceptable, one journal could offer to take the lead in publishing it as a full document, while the other could publish the executive summary.	Not mentioned
20-30	• An article was published by a researcher in a journal. Two months later, another independent researcher demanded retraction due to minor actual contribution without any new experimental data. • The author responded stating that neither the researcher met the minimum set of standards for professional behavior nor based on solid grounds. • The researcher states that they have a “veto” power and can prohibit publications from the collaboration. • Journal felt that the researcher's concern was beyond the prerogatives of the journal and decided not to retract but offered opportunities to both the researcher and author to comment and reply, respectively on the published article. • An independent referee suggested modifications to the comment from the researcher to make it professional.	• The journal's response was appropriate. • There is no necessity to publish expressions of concern/corrigendum or to retract. • The journal should request the researcher to modify the comment based on the independent referee's review. • In case the journal wishes to follow up on the veto power of the researcher, they must ask for an appropriate proof. • The journal should also inform the relevant institutions about this dispute.	Not mentioned

ICMJE, international council of medical journal editors; and EIC, editor in chief.

### Selection of LLMs and prompting strategies

Two open-source LLMs, Google Gemini 2.5 Flash and DeepSeek-V3.2, were specifically selected for this evaluation as they represent the leading edge of open-source and high-throughput LLM architecture, featuring advanced native reasoning capabilities and expansive context windows. These models were chosen over others to provide a rigorous comparison between a sparse Mixture-of-Experts (MoE) transformer optimized for multimodal reasoning (Gemini) and a high-parameter MoE framework designed for extreme computational efficiency and advanced chain-of-thought processing (DeepSeek).

Three types of prompts (Lee and Shin, 2024) were used with each of the above LLMs:

- Minimal prompt: to assess the “raw” model behavior with minimal steering (Electronic Supplementary File 1). Only basic roles, tasks and the exact COPE case were used for eliciting response.
- Prompt for deterministic response (Electronic Supplementary File 2): in addition to the roles, tasks and contexts, additional instructions and constraints along with the exact COPE case were used for eliciting response.
- Prompt for stochastic response (Electronic Supplementary File 3): in addition to the prompts used for deterministic response, additional instruction (“you may explore alternative reasonable editorial approaches”) was added providing the model room to vary emphasis, thus allowing an opportunity for randomness in their expression.

No changes were made in parameter settings to reflect the performance of LLMs in real-world settings. The initial output generated by both Gemini 2.5 Flash and DeepSeek-V3.2 consisted of unstructured text in the form of narrative editorial advice and step-by-step recommendations. No log-probabilities or token-level classification scores were extracted during the inference phase. In the post-processing stage, these unstructured narrative responses were converted into structured quantitative data for analysis. This transformation involved two independent raters who mapped the qualitative advice onto a standardized 5-point Likert scale across seven evaluative domains.

### Multi-domain evaluation rubric

Each of the two authors independently assessed LLM responses with a structured, predefined rubric adapted to the ethical reasoning framework of the COPE (Electronic Supplementary File 4). Each response was independently assessed across seven domains: (1) fidelity to the COPE forum perspective, (2) identification of ethical issues, (3) actionability of recommendations, (4) consistency with COPE principles, (5) safety and avoidance of hallucination, (6) clarity, structure, and readability, and (7) overall appropriateness of the recommendation. Fidelity to the COPE forum perspective assessed the degree to which the LLM's conclusion and recommended course of action aligned with the specific advice and consensus reasoning provided by COPE forum members for the anonymized case in question. A high score indicated that the LLM captured the core recommendation and rationale from the expert discussion. Consistency with COPE principles evaluated whether the LLM's response was grounded in the broad, codified guidelines and ethical standards published by COPE. To ensure the construct validity of our evaluation and to ground the rubric directly in the COPE framework, the seven domains were derived from the hypothesized COPE's decision-making protocol that should typically involve characterizing the ethical dilemma based on submitted facts, identifying relevant ethical principles and guidelines, formulating a stepwise, practical course of action for the editor, and ensuring all advice is prudent, avoids harm, and is communicated clearly to facilitate implementation. Rubric operationalizes this protocol into measurable domains as follows:

- Fidelity to the COPE forum perspective and identification of ethical issues correspond to the initial, critical steps of case comprehension and issue spotting, ensuring the model correctly interprets the scenario's core ethical conflict.
- Consistency with COPE principles assesses the model's ability to reference and apply relevant COPE guidelines and broader publication ethics norms.
- Actionability of recommendations and clarity, structure, and readability translate COPE's advisory role into concrete, implementable steps communicated in an editorially useful format.
- Safety and avoidance of hallucination is a paramount domain reflecting COPE's commitment to responsible, evidence-based guidance, penalizing the generation of fictitious policies or risky advice.
- Overall Appropriateness serves as a holistic judgment mirroring the COPE forum's final synthesis, evaluating whether the collective advice is balanced, proportionate, and fit-for-purpose.

Each domain was rated on a 5-point ordinal Likert scale (1 = very poor, 2 = poor, 3 = fair, 4 = good, 5 = excellent), with higher scores indicating closer alignment with COPE-style editorial reasoning and safer, more actionable guidance. Domain-level scores were analyzed individually and aggregated into a composite score representing overall response quality and were consistently applied for each of the cases. Each domain was given equal weightage considering their critical contributions. The rubric was developed through iterative discussions with the authors, who drew on collective expertise in serving as an Editorial Board member of a peer-reviewed journal, peer reviewing for over 400 articles, and conducting courses on research ethics. These metrics are highly relevant to the downstream task of editorial triaging, as they directly measure a model's ability to provide safe, actionable, and COPE-aligned advice. To ensure the validity of these AI-generated outputs for their intended use, the metrics were subjected to human evaluation by independent raters. The correlation between the automated text generation and human ethical standards was quantified through similarity scores compared against gold-standard COPE forum conclusions, ensuring that the quantitative metrics accurately reflect the qualitative depth and safety required for resolving authorship disputes. Three pilot cases were used for calibrating the rubric that was applied independently by the raters. The IRR was quantified using Cohen's kappa, and discrepancies were resolved through consensus.

### Concordance and hallucination assessment

In addition to domain-based quality scoring, each LLM response was evaluated for concordance with the corresponding COPE expert response (Electronic Supplementary File 5). Concordance was assessed using a multi-component framework that captured both overall similarity and specific areas of agreement or deviation. First, raters scored the similarity between the LLM's conclusion and the COPE conclusion on a 5-point ordinal scale (1 = very low similarity to 5 = very high similarity). Second, the overall level of agreement was categorized as fully agrees, partially agrees, minor disagreement, major disagreement, or contradictory. Raters also recorded whether any critical elements present in the COPE response were missing from the LLM output and whether the LLM included any inappropriate or potentially misleading advice. To explicitly address safety and factual reliability, a structured hallucination checklist was applied, documenting whether the LLM invented non-existent COPE policies, fabricated allegations or misconduct details, introduced fictitious individuals, or provided fabricated references. Any additional hallucinations not captured by predefined categories were documented in free text. These concordance and hallucination measures were analyzed alongside rubric-based scores to provide a comprehensive assessment of fidelity, agreement, and safety relative to COPE forum guidance.

### Statistical analysis and reporting standards

Statistical analyses were performed using R version 4.5.1, with numerical variables reported as mean ± SD. Normality was assessed via the Kolmogorov-Smirnov test. The Mann-Whitney U test was used to compare scores between the two LLMs, while the Kruskal-Wallis H test was employed to analyze similarity scores across different prompt types. Categorical variables were evaluated using Fisher's exact probability test, and *p*-values < 0.05 were considered statistically significant. Data visualization included heatmaps for domain scores, violin plots for similarity distribution, and box plots for prompt-based stability analysis. This study employed an observational design using a complete set of cases from a defined cohort rather than a sample sized to detect a specific effect. Therefore, a formal *a priori* power calculation was not conducted. The sample size of 12 cases inherently limits the statistical power to detect anything but large differences between groups. This study is reported in compliance with the TRIPOD-LLM reporting requirements for studies evaluating large language models in healthcare and research (Gallifant et al., 2025).

## Results

### Overview of model responses

Both Google Gemini 2.5 Flash and DeepSeek-V3.2 provided comprehensive and complete responses to all 12 authorship and contributorship case scenarios. Overall IRR was observed to be substantial (Cohen's kappa = 0.76). Full details of the specific prompts used, and the corresponding raw model outputs are available in Electronic Supplementary File 6.

### Comparative model overall performance

**Figure 1:**
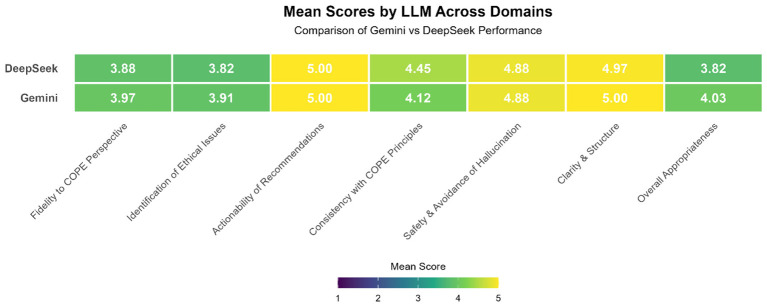
Comparative evaluation of Gemini and DeepSeek LLM performance across ethical and quality domains. This heatmap illustrates the mean performance scores for the Gemini and DeepSeek LLMs across seven evaluative categories. These domains include fidelity to COPE forum perspectives, identification of ethical issues, actionability, safety, and structural clarity. Scores are measured on a 1–5 scale, with higher values (represented by yellow-toned cells) indicating superior performance.

The comparative analysis of model performance across seven evaluative domains reveals that both Gemini and DeepSeek exhibit exceptional proficiency in high-stakes operational metrics (Figure 1). Both models achieved perfect scores (5) in Actionability of Recommendations, alongside high marks for Safety & Avoidance of Hallucination (4.88), indicating a shared capability for producing reliable and implementation-ready outputs. However, distinct performance variations emerged in domain-specific alignment and structural quality. DeepSeek demonstrated slight advantage in consistency with COPE principles (4.45 vs. 4.12), suggesting a stronger adherence to standardized ethical frameworks. Conversely, Gemini consistently outperformed DeepSeek slightly both in clarity and structure (5 vs. 4.97) and overall appropriateness (4.03 vs. 3.82). Both models registered their lowest performance levels in fidelity to COPE forum perspective and identification of ethical issues, where scores ranged from 3.82 to 3.97. While formal statistical comparisons did not reach significance (*p* > 0.05), likely due to limited power, the observed trends and effect sizes suggest that while both LLMs are technically proficient and safe, they face shared challenges in navigating the nuanced subjective perspectives and complex ethical underpinnings required by the COPE framework.

### Impact of prompt types on model performance

**Figure 2:**
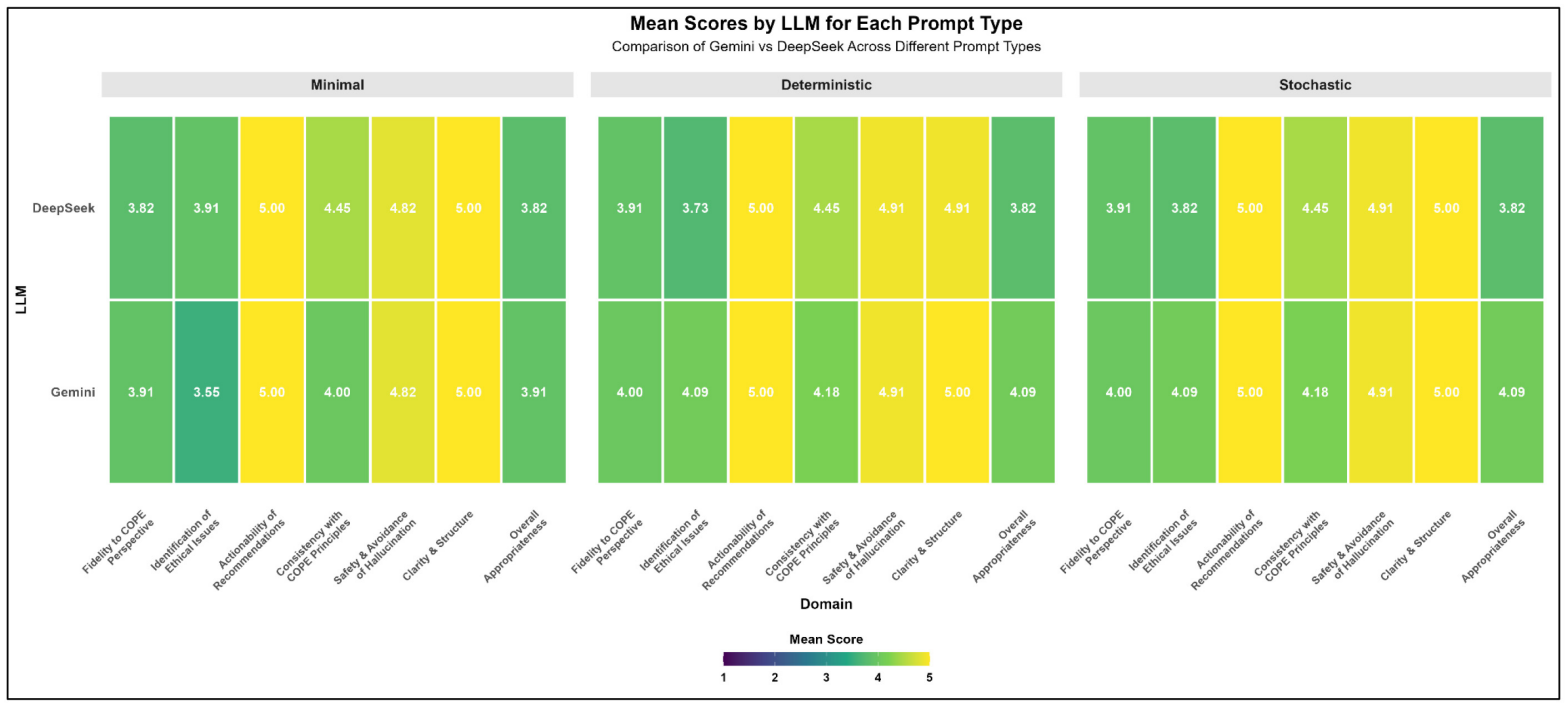
Comparative performance of Gemini and DeepSeek LLMs across prompt strategies and evaluative domains. This faceted heatmap displays the mean performance scores for Gemini and DeepSeek across seven evaluative domains, segmented by prompt type: minimal, deterministic, and stochastic. Scores are measured on a scale of 1–5, with higher values (Yellow) indicating superior alignment with evaluative criteria.

The stratified analysis across Minimal, Deterministic, and Stochastic prompting strategies reveals distinct patterns of model stability and sensitivity (Figure 2). Both Gemini and DeepSeek exhibited robust performance in actionability of recommendations (5) and safety and avoidance of hallucination (4.82–4.91) regardless of the prompt type, suggesting these capabilities are deeply embedded in the models' core logic. However, Gemini demonstrated significant sensitivity to prompt complexity; its scores for identification of ethical issues improved markedly from a study-low of 3.55 under Minimal prompts to 4.09 in both Deterministic and Stochastic conditions. A similar upward trend for Gemini was observed in overall appropriateness, rising from 3.91 to 4.09 (Table 2). In contrast, DeepSeek displayed remarkable performance stability across all three facets, maintaining identical scores for consistency with COPE principles (4.45) and overall appropriateness (3.82) across all prompting conditions. While DeepSeek consistently outperformed Gemini in consistency with COPE principles, Gemini maintained a superior edge in clarity and structure and overall appropriateness when provided with more detailed (Deterministic or Stochastic) instructions. These findings indicate that while DeepSeek offers higher reliability and consistency regardless of prompt engineering, Gemini's performance, particularly in nuanced ethical identification, is highly contingent upon the specificity and nature of the input prompt.

**Table 2:** Comparisons of overall and domain scores between the LLMs.

Domain	Google Gemini			DeepSeek			*P*-values
Fidelity to COPE forum perspective	3.97 ± 1.36			3.88 ± 1.32			0.62
Identification of ethical issues	3.91 ± 1.33			3.82 ± 1.29			0.76
Actionability of recommendations	5 ± 0			5 ± 0			NA
Consistency with COPE principles	4.12 ± 1.29			4.45 ± 1			0.28
Safety and avoidance of hallucination	4.88 ± 0.33			4.88 ± 0.33			1
Clarity and structure	5 ± 0			4.97 ± 0.17			0.33
Overall appropriateness	4.03 ± 0.98			3.82 ± 1.29			0.71
Prompt types	M	D	S	M	D	S	
Fidelity to COPE forum perspective	3.9 ± 1.5	4 ± 1.34	4 ± 1.34	3.8 ± 1.47	3.9 ± 1.3	3.9 ± 1.3	0.8; 0.8; 0.8
Identification of ethical issues	3.55 ± 1.44	4.1 ± 1.3	4.1 ± 1.3	3.9 ± 1.38	3.73 ± 1.35	3.82 ± 1.25	0.5; 0.5; 0.6
Actionability of recommendations	5 ± 0						NA
Consistency with COPE principles	4 ± 1.34	4.18 ± 1.33	4.19 ± 1.33	4.45 ± 1.04	4.45 ± 1.04	4.45 ± 1.04	0.4; 0.66; 0.66
Safety and avoidance of hallucination	4.82 ± 0.4	4.91 ± 0.3	4.91 ± 0.3	4.82 ± 0.4	4.91 ± 0.3	4.91 ± 0.3	NA
Clarity and structure	5 ± 0				4.91 ± 0.3	5 ± 0	NA
Overall appropriateness	3.91 ± 0.94	4.09 ± 1.04	4.09 ± 1.04	3.82 ± 1.33	3.82 ± 1.33	3.82 ± 1.33	0.95; 0.73; 0.73

*P*-values were estimated using Mann-Whitney U test; M-minimal prompt; D-deterministic prompt; S-stochastic prompt; NA: not applicable.

### Qualitative analysis and alignment with COPE members responses

**Figure 3:**
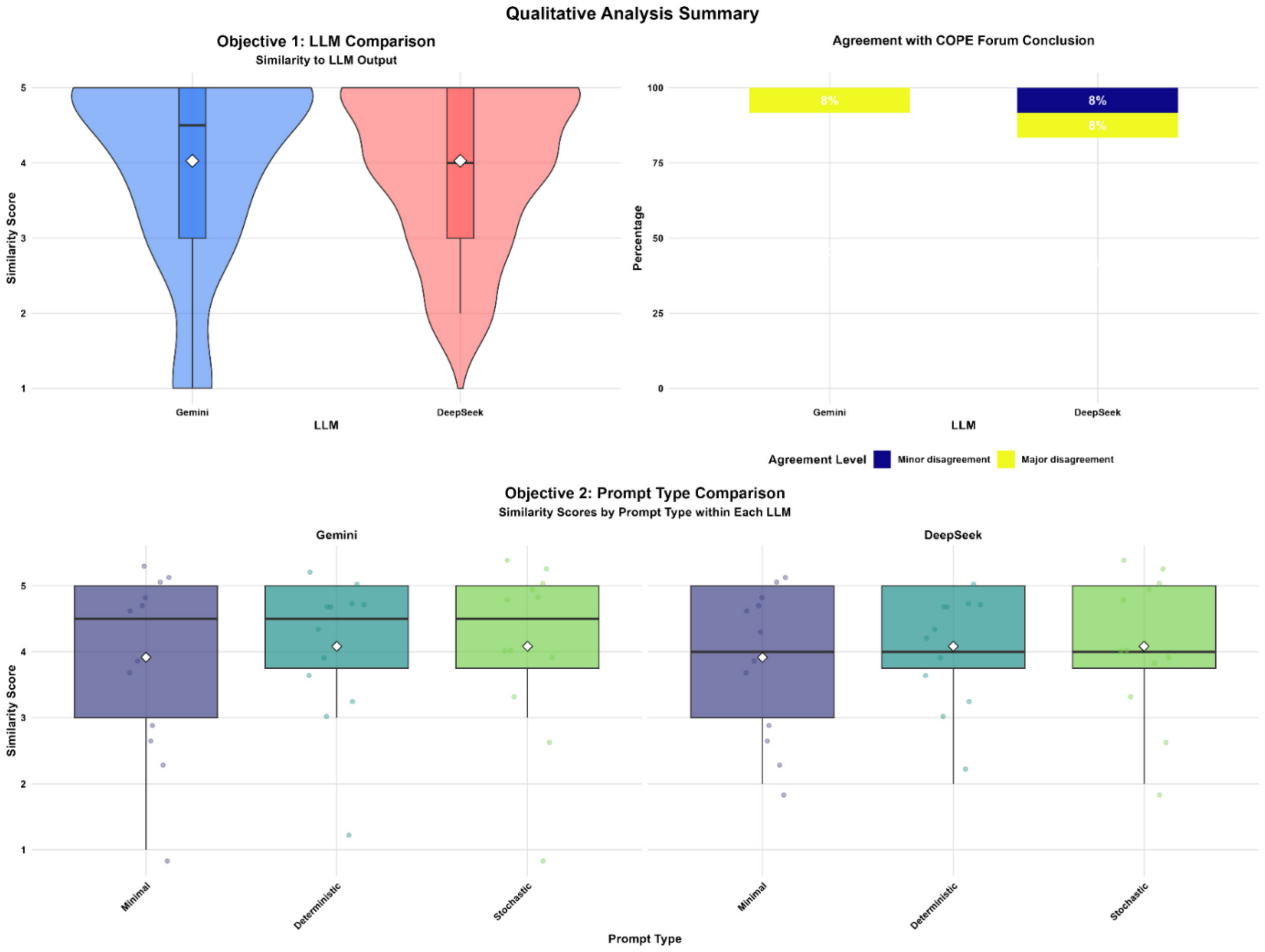
Qualitative evaluation of model similarity and alignment with COPE forum conclusions. This multi-panel figure provides a qualitative summary of LLM outputs compared to COPE member responses. Top Left: violin plots illustrate the kernel density and distribution of similarity scores for Gemini and DeepSeek, with white diamonds indicating the mean score. Top Right (agreement level): a stacked percentage chart shows the frequency of model disagreement with COPE Forum conclusions. The legend indicates the severity of disagreement: major disagreement (Yellow), and minor disagreement (Dark Blue). Bottom: box plots depict the distribution of similarity scores within each model, stratified by prompt type (minimal, deterministic, and stochastic), highlighting the relative stability of model performance across different input styles.

The qualitative evaluation (Figure 3) further confirmed the high degree of alignment between the models and expert standards. When evaluated against the COPE Forum conclusions, both models demonstrated a high rate of consensus. Gemini recorded an 8% rate of major disagreement, while DeepSeek exhibited a combined 16% disagreement rate, consisting of 8% minor and 8% major disagreements. The impact of prompting strategies on qualitative similarity scores mirrored the trends observed in the quantitative domain analysis. For both Gemini and DeepSeek, mean similarity scores improved from 3.9 under Minimal prompting to 4.1 when using Deterministic or Stochastic prompts. While both models benefited from more detailed instructions, DeepSeek demonstrated greater distributional stability across prompt types. In contrast, Gemini's outputs under Minimal prompting showed a wider range of variance, suggesting that while it can reach high similarity peaks, its performance is more sensitive to the structural quality of the input.

### Comparison of LLM responses across individual cases

The comparative analysis revealed that while both Gemini and DeepSeek demonstrate a high degree of conceptual alignment with COPE principles, they consistently offered supplementary “value-add” recommendations not explicitly found in original COPE responses (Table 3). Detailed assessment of the LLM responses across each case and the prompt type can be found in the Electronic Supplementary File 7. Across all 12 cases, both models frequently missed specific legal or administrative nuances, such as checking for copyright infringement (Case 20-07), verifying publishing agreements (Case 20-11), backing up retraction should not be solely based on the authorship issues (Case 20-07), or contacting secondary institutions (Case 20-15), understanding the potential authors' dimensions (Case 20-08), and it is incorrect to provide unsolicited information to an editor of another journal (Case 20-20) that are central to the COPE forum's expert advice. However, the models compensated for these omissions by providing actionable, unique strategies (indicated by asterisks in the data), including the development of de-escalation training for editors (Case 20-08), developing a journal policy on code of conduct for authors (Case 20-08), recommending reinstating the retracted article by the editor (Case 20-10), internal flagging systems for non-cooperative authors (Case 20-11, 20-23), identifying the existing journal policy and conflicts between the authors request (Case 20-25), and the proposal of “Author Disassociation Statements” for professional misconduct scenarios (Case 20-17).

**Table 3:** Comparative analysis of Gemini and DeepSeek responses in authorship and contributorship case scenarios across prompting strategies.

Case No.	Prompt type	Google Gemini 2.5 flash	DeepSeek-V3.2
20-07	Minimal	Missed COPE retraction/legal points; explicitly labeled scientific misconduct and suggests potential sanctions^*^.	Missed COPE retraction/legal points; clear steps for resubmission.
	Deterministic/Stochastic	Outlined formal documentation steps; suggested co-author automated confirmation^*^.	Focus on verifying co-author stances; missed specific COPE policy update advice.
20-08	Minimal	Suggested journal policy on code of conduct for authors^*^ and documenting unprofessional behavior.	Suggested editor's de-escalation training^*^; proposed structured appeal responses.
	Deterministic/Stochastic	Recommended consulting legal for freedom of information; journal policy on code of conduct for authors^*^.	Suggested editor's de-escalation training^*^; focus on maintaining records and citing author code of conduct; journal policy on code of conduct for authors and internal social media check^*^.
20-10	Minimal	Encouraged resubmission with DOI disclosure; publisher article reinstatement^*^.	Highlighted unfairness to other authors; suggested article expansion/transformation^*^.
	Deterministic/Stochastic	Issued editorial note for reinstatement; identified inflexible institutional policies^*^.	Recommended Crossref clarification on withdrawal policies for non-ethical reasons^*^.
20-11	Minimal	Final letter to all emails; identified high risk in publishing without final proof; suggests rare instance for publishing manuscripts where authors could not be contacted^*^.	Willing to discuss resubmission after new assessment; missed legal/copyright points.
	Deterministic/Stochastic	Recommended flagging non-cooperative authors internally^*^; emphasize author rights.	Suggested creating a formal unresponsive author policy; missed legal/copyright points.
20-15	Minimal	Detailed content for notice of concern; missed legal/second institution contact.	Identical to Gemini; detailed notice content; missed legal/second institution contact.
	Deterministic/Stochastic	Verification of institutional appeal completion; determined findings' impact on findings.	Investigated if institutional findings relate to paper; escalate to retraction if public evidence^*^.
20-17	Minimal	Suggested “Author Disassociation Statement”^*^; accountability via CRediT criteria; redesignation of coauthor as contributor^*^.	Removal only after formal institutional findings; reclassify as contributor.
	Deterministic/Stochastic	Warned against precedent of removing authors for reputational damage^*^; consult legal.	Like Gemini; specified lack of mandate to alter record for professional conduct; redesignation of coauthor as contributor^*^.
20-18	Minimal	Noted phone calls as harassing^*^; obtain evidence of contribution before referral.	Advised status quo until institutional finding; suggested temporary editorial note^*^.
	Deterministic/Stochastic	Suggested Joint Authorship Agreement before referring to institution and creating author dispute policy^*^.	Requested joint authorship agreement before referral^*^; Drafted temporary editorial note^*^.
20-20	Minimal	Banned future submissions if unverifiable^*^; internal ethics audit.	Suggested note of concern; verified documentation matched affiliations.
	Deterministic/Stochastic	Consulted legal counsel; labeled harassment as professional misconduct^*^.	Suggested fetching additional documentary evidence (screenshots) from other journals^*^.
20-23	Minimal	Avoid parallel investigation if formal one exists^*^; internal author group flagging^*^.	Proposed sharing rejection details with consortium journals^*^; monitor situation.
	Deterministic/Stochastic	Provided specific rejection template citing data integrity concerns^*^.	Allowed opportunity for authors to revert after external investigation ends^*^.
20-25	Minimal	Identified violation of late-stage authorship policy^*^; noted gift authorship suspicion.	Like Gemini; suggested withdrawal if authors disagree with rejection.
	Deterministic/Stochastic	Cited disruption of editorial process integrity^*^; justified as administrative/logistic.	Offered publication with original names or rejection if new author insisted; peer review and revisions conducted with understanding of the original authors^*^.
20-29	Minimal	Noted waste of resources via redundant publication^*^; secondary version “reprint” route.	Proposed neutral third journal^*^; missed COPE's collective group authorship solution.
	Deterministic/Stochastic	Listed translation/republication exceptions; insisted on single journal publication.	Identified ambiguity for readers and citations if multiple versions existed.
20-30	Minimal	Final opportunity for professional editing of comments^*^; pause until institutional finding.	Agreed with journal action; missed proof requirement for researcher “veto power”.
	Deterministic/Stochastic	Stated veto rights are institutional agreements, not publication ethics^*^.	Evaluated net scholarly value of comment vs. non-scholarly contentious purpose^*^.

^*^Identified exclusively by the LLM and not present in the original COPE forum response.

Between the models, Gemini often displayed a more assertive stance on research integrity, explicitly labeling actions as “scientific misconduct” and suggesting potential sanctions (Case 20-07), recommending the publisher reinstate the retracted article (Case 20-10) and explicitly stated that the publisher made serious ethical error in retracting the article (Case 20-10) where DeepSeek remained neutral. Gemini also provided more elaborate procedural frameworks for reinstating retracted articles, including reactivating DOI (Case 20-10), stated that veto rights are not publication ethics (Case 20-30) and managing harassment (Case 20-18). DeepSeek, with a similar proficiency, showed remarkable strengths in suggesting structured appeal responses (Case 20-08), fetching additional details for verifying authors affiliations (Case 20-20), and maintaining a consistent focus on institutional verification for questionable affiliations (Case 20-20) and additionally suggested social media verification for ensuring that the authors' poll does not misrepresent journal/editor/process (Case 20-08), and peer review and revisions of a manuscript are conducted with understanding of a specific author team that can be impacted by the request for adding authors following acceptance as well insists withdrawal of the manuscript in case the authors insist (Case 20-25). In one instance, DeepSeek provided risky advice adding a temporary editorial note for an authorship issue related to statistical analysis contribution in the absence of any written agreement (Case 20-18). In complex cases like 20-29 (redundant guidelines), both models failed to identify the specific COPE solution of collective group authorship, instead prioritizing the enforcement of single-journal publication to avoid resource waste.

Prompt engineering significantly influenced the depth and quality of the outputs. Minimal prompts frequently led to the omission of critical COPE-aligned advice, such as stating that rejection of a manuscript without investigating the authors' affiliation where it is questionable as appropriate (Case 20-20). Furthermore, Minimal prompt (with Gemini) provided potentially unethical and risky advice such as considering publication of a manuscript addressing immediate and significant public health problems when the author/s could not be contacted (Case 20-11) and considering redesignation of a coauthor following publication of an article as a contributor for a non-scientific and unethical reason (Case 20-17). In contrast, Deterministic and Stochastic prompts consistently elicited higher-order reasoning, producing detailed step-by-step documentation sequences (Case 20-07), laying down criteria where the same manuscript from consensus guidelines can be published in more than one journal (Case 20-29), fetching additional details for verifying authors affiliations (Case 20-20), specific communication templates for authors and institutions (Case 20-18, 20-23), and more nuanced ethical justifications such as obtaining coauthors' stance (Case 20-07), and author additions after acceptance of a manuscript should not be encouraged as the peer review and revisions are carried out with the understanding of original authors (Case 20-25). However, in only one instance, DeepSeek, for both Deterministic and Stochastic prompts, provided potentially incorrect advice on the redesignation of a coauthor following publication of an article as a contributor for a non-scientific and unethical reason (Case 20-17) that was not observed with minimal prompt. Additionally, Stochastic prompt suggested providing an additional opportunity for authors to revert after external investigation is completed (Case 20-23) and stated that veto rights do not constitute publication ethics (Case 20-30). Despite these minimal variations, the Stochastic variability did not consistently resolve the models shared blind spot regarding the legal implications of signed publishing agreements, indicating a persistent gap in LLM understanding of the formal legalities of scholarly publishing. However, in few cases, stochastic prompts generated useful recommendations not observed with either minimal or deterministic types (with Gemini) such as informing the unprofessional conduct of the author to coauthors (Case 20-08) and suggesting having a signed Joint Authorship Agreement before institutional referral (Case 20-18).

## Discussion

### Key findings

This study demonstrated that LLMs, specifically Gemini 2.5 Flash and DeepSeek-V3.2, possess capacity for navigating complex ethical dilemmas in authorship and contributorship, achieving high alignment with expert COPE forum responses. Our findings reveal that while both models are exceptionally reliable in terms of safety, structural clarity, and the actionability of their recommendations, they exhibit a consistent “legal blind spot” regarding the formal administrative and copyright nuances inherent in scholarly publishing. A pivotal discovery of this research is the differing nature of model stability: DeepSeek showed robustness across all prompting strategies, whereas Gemini's performance, particularly in identifying deep-seated ethical issues, was significantly enhanced by deterministic and stochastic prompt engineering. Furthermore, the models did not merely replicate expert advice but acted as “value-add” contributors by proposing novel strategies, such as author disassociation statements and editorial de-escalation training, which were absent from the original COPE forum responses. Collectively, these results suggest that while LLMs cannot yet replace the nuanced legal and institutional oversight of expert committees, they have matured into highly effective supplementary tools for preliminary ethical screening and the generation of structured editorial frameworks.

### Comparison with existing literature

Authorship has gained unprecedented significance in academia, directly influencing personal professional satisfaction and the appointment or promotion of faculty members (Tarkang et al., 2017). However, unethical authorship practices, including coercive, gift, and ghost authorship, are becoming increasingly prevalent; recent estimates suggest such issues may affect up to one-third of the scientific literature within the European Union (Aliukonis et al., 2020). While these disputes remain among the most difficult for publishers to resolve, recent transformations in digital submission systems and a global drive toward transparency have become instrumental in mitigating these challenges (Anstey, 2014). The present study is the first to evaluate the utility of LLMs in assisting journal editors with ethical dilemmas related to authorship and contributorship, using expert COPE member responses as a benchmark. Our findings indicate that both Gemini and DeepSeek can identify most ethical issues presented in real-world scenarios. This aligns with recent research assessing ChatGPT's performance on medical ethics, which reported an overall score of 4.1/5, with high marks for technical (4.7/5) and non-technical clarity (4.4/5; Balas et al., 2024). However, as noted by researchers in that study, further improvements are required for AI to master the depth of complex, nuanced ethical scenarios (Balas et al., 2024). We observed a similar trajectory in our results, with Gemini and DeepSeek achieving mean scores of 3.97/5 and 3.88/5, respectively, in fidelity to the COPE forum perspective. Because the models used in this study were not specifically trained in journal management or publication ethics, their ability to make highly nuanced judgments is inherently limited. Implementing fine-tuning strategies, particularly Retrieval-Augmented Generation (RAG), is likely to enhance accuracy and further mitigate the risk of hallucination (Mann et al., 2025). We also observed that despite the use of sophisticated deterministic and stochastic prompting, certain “blind spots” persisted. This phenomenon is a known characteristic of LLMs and is primarily attributed to limitations within their training datasets (Soffer et al., 2025). This likely explains why specific legal nuances were overlooked by both models in this study.

Nevertheless, the high safety scores achieved by both models under deterministic and stochastic prompting conditions indicate a strong potential for reliable assistance when used with appropriate safeguards. However, it is crucial to distinguish this optimized performance from unstructured use. As our results show, minimal prompting led to instances of incorrect or hazardous advice (e.g., Case 20-11, 20-17), demonstrating that unsupervised, off-the-shelf implementation carries significant risk. Therefore, claims of model “safety” and “readiness” are strictly contingent upon the use of carefully engineered prompts and robust human oversight. This high reliability in ‘safe' reasoning under structured prompting suggests editors can delegate certain time-intensive tasks to AI as a preliminary screening layer. However, this delegation must be predicated on two non-negotiable conditions: (1) the use of deterministic, role-constrained prompts to steer the model away from the risks observed with minimal prompting, and (2) the maintenance of a strict “human-in-the-loop” verification process where an expert editor critically reviews all AI-generated advice before any action is taken. Unsupervised implementation is not currently advisable. Alternatively, focusing on training LLMs predominantly within the relevant domain has been shown to be an effective strategy for mitigating hallucination risk (Zhang et al., 2025).

Crucially, LLMs should be viewed as an augmentation of, rather than a replacement for, human expertise. In our study, the models provided “value-add” strategies, such as “Author Disassociation Statements” and de-escalation training for editors, that were entirely absent from the original COPE forum responses. These additional contributions demonstrate that LLMs can offer supplementary perspectives and rapid structural frameworks for communication. However, human oversight remains paramount to ensure these recommendations are applied with the necessary empathy and contextual intuition, as overreliance on AI carries risks of embedded bias (Carnat, 2024).

The persistent “legal blind spot” regarding copyright infringement and publishing agreements likely stems from the fact that while LLMs have extensive access to public ethical frameworks, they lack visibility into the proprietary, non-public legal contracts governing author-publisher relationships (Wallat et al., 2024). To overcome this, we recommend the development of localized LLMs trained specifically on publication ethics guidelines, legal frameworks, and the extensive repository of COPE discussion forum cases. Such localized AI systems would also serve to safeguard the privacy of sensitive editorial responses (Huang et al., 2024).

Finally, this study highlights differences between the LLMs on their stability depending on the prompt type. While DeepSeek-V3 exhibited remarkable stability across all conditions, Gemini 2.5 Flash demonstrated profound sensitivity to prompt engineering; its ability to identify ethical issues improved significantly from a study-low of 3.55 under minimal prompts to 4.09 under deterministic and stochastic conditions. Gemini's architecture may require more robust, multi-step instructions to activate its reasoning logic, whereas DeepSeek's MoE framework appears better calibrated for consistent factual extraction regardless of prompt depth. These differences may be attributed to variations in Reinforcement Learning from Human Feedback (RLHF; Christiano et al., 2017), as well as specific augmentation and instruction-tuning methods (Chai et al., 2026; Zhang et al., 2023). Given these variations, it is prudent for editors and researchers to utilize multiple LLMs rather than a single model to ensure optimal and balanced editorial responses.

### Strengths, limitations and way forward

The primary strength of this research lies in its rigorous, multi-dimensional evaluation framework that bridges the gap between qualitative ethical reasoning and quantitative performance metrics. By utilizing real-world, anonymized cases from the COPE forum, the study ensures that the LLMs were tested against high-fidelity, peer-validated benchmarks rather than synthetic or oversimplified scenarios. The inclusion of three distinct prompting strategies, minimal, deterministic, and stochastic, provides a sophisticated understanding of model stability and sensitivity, offering rare insights into how “raw” vs. “steered” AI behavior impacts ethical alignment. Furthermore, the dual-model comparison between Gemini and DeepSeek highlights how different architectural philosophies, such as hybrid reasoning and large-scale MoE frameworks, handle the nuance of authorship disputes. The prospective registration of the protocol and adherence to TRIPOD-LLM reporting standards further enhance the transparency, reproducibility, and methodological integrity of the findings.

Despite its robust design, several limitations must be acknowledged. First, the scope of this analysis was intentionally focused but inherently limited. The evaluation was restricted to 12 cases from the “authorship and contributorship” domain within a single year. While this provided a controlled, in-depth examination of a high-prevalence ethical category, it necessitates caution regarding generalizability. Our findings on model proficiency in navigating interpersonal disputes and credit attribution may not directly translate to other COPE domains, such as plagiarism, data fabrication, or peer-review manipulation. These domains often involve different cognitive tasks, such as factual verification, forensic analysis of data/images, or detecting complex fraud patterns, where an LLM's capability might differ. Furthermore, the case set did not include contemporary dilemmas involving AI-generated content, which presents a novel, meta-ethical challenge for publication ethics. The performance of LLMs in scenarios where they must adjudicate the use or disclosure of their own kind of technology remains an untested and critical area for future research. Second, the evaluation relies on a 5-point Likert scale and human raters, which, although calibrated and measured for inter-rater reliability, still introduces a degree of subjective interpretation regarding what constitutes “fidelity” to the COPE forum perspective. To mitigate the risks of confirmation bias and role conflict inherent in having authors serve as raters, several procedural safeguards were implemented. First, the raters completed calibration sessions using three pilot cases to establish a shared understanding of the domain criteria and scale anchors before evaluating the study cases. Second, all evaluations were performed independently and privately prior to any comparative discussion. Third, inter-rater reliability was quantified using Cohen's kappa to ensure objective measurement of agreement. Finally, any scoring discrepancies were resolved through a consensus discussion focused strictly on the rubric criteria and the textual evidence from the COPE response. Although the structured protocol aimed to anchor ratings to specific criteria, the lack of blinding represents a potential source of bias. Additionally, this study evaluated the models at a specific point in time; the rapid iterative nature of LLM development means that subsequent model updates might yield different results. Also, the sample size of 12 cases, while comprising the relevant population for the studied year and domain, limits the statistical power of the study. This constrains our ability to detect anything but large effect sizes in the comparisons between models or prompt types and increases the uncertainty around point estimates. As noted, our interpretation therefore appropriately prioritizes effect magnitude and patterns over binary statistical significance, but future studies with larger case sets would allow for more precise statistical inference. Finally, the “legal blind spot” observed, specifically regarding copyright and publishing agreements, highlights a fundamental limitation in the models' training data, which often lacks access to proprietary or confidential legal contracts between authors and publishers.

The results of this study suggest a transformative way forward for the integration of AI in scholarly publishing. For journal editors, LLMs should be viewed as “intelligent assistants” rather than autonomous decision-makers. They are highly effective for the preliminary triaging of ethical disputes, drafting structured communication templates, and identifying potential “value-add” strategies like de-escalation training or internal flagging systems. Editors are encouraged to use deterministic prompting, providing clear roles and constraints, to maximize the reliability of these tools. For researchers, these findings offer a cautionary but optimistic roadmap; LLMs can provide immediate, actionable feedback on best ethical practices during the manuscript preparation phase, but they must be secondary to established COPE and institutional guidelines. Moving forward, the development of “domain-specific” LLMs, fine-tuned on comprehensive legal and ethical publishing datasets, could bridge the existing gap in administrative and legal nuances. Future research should focus on longitudinal assessments to track model improvement and explore the use of multi-agent AI systems, where different models “debate” ethical cases to arrive at a consensus even closer to expert human judgment.

## Conclusion

In conclusion, this study demonstrates that LLMs such as Gemini 2.5 Flash and DeepSeek-V3.2 have attained a high degree of proficiency in navigating complex ethical dilemmas within the authorship and contributorship domain. Both models exhibit a remarkable alignment with the expert reasoning provided by the COPE forum, particularly when optimized through structured deterministic and stochastic prompting strategies. While the models demonstrate near-perfect performance in generating safe, clear, and actionable editorial recommendations, they possess a distinct “legal blind spot” regarding the formal administrative and copyright nuances of scholarly publishing. Furthermore, the ability of LLMs to offer “value-add” perspectives, proposing unique solutions like author disassociation statements and de-escalation frameworks, indicates their potential to expand the toolkit available to journal editors. While this study establishes a high baseline of LLM competency for authorship and contributorship ethics, their applicability as editorial tools should be validated domain-by-domain. Future research must test these models against cases of plagiarism, data integrity, and the unique complexities introduced by AI-generated scholarly content to fully map their capabilities and limitations across the entire spectrum of publication ethics. Ultimately, while AI cannot replace the specialized oversight of expert committees and legal counsel, it can, when used with deliberately engineered prompts and expert human verification, serve as a powerful and consistent supplementary tool. Its reliability is not inherent but is a function of constrained implementation. This study formally cautions against unsupervised use and positions advanced LLMs as potential aids for enhancing editorial workflow efficiency, provided their integration is guided by structured prompting and upheld by definitive human editorial judgment.

## Data Availability

The original contributions presented in the study are included in the article/Supplementary material, further inquiries can be directed to the corresponding author.

## References

[B1] Aliukonis V. Poškute M. Gefenas E. (2020). Perish or publish dilemma: challenges to responsible authorship. Medicina 56:123. doi: 10.3390/medicina56030123 32178434 PMC7142498

[B2] Alkalbani A. M. Alrawahi A. S. Salah A. Haghighi V. Zhang Y. Alkindi S. . (2025). Systematic review of large language models in medical specialties: applications, challenges and future directions. Information 16:489. doi: 10.3390/info16060489

[B3] Anstey A. (2014). Authorship issues: grizzles, guests and ghosts. Brit. J. Dermatol. 170, 1209–1210. doi: 10.1111/bjd.13095 24947146

[B4] Balas M. Wadden J. J. Hébert P. C. Mathison E. Warren M. D. Seavilleklein V. . (2024). Exploring the potential utility of AI large language models for medical ethics: an expert panel evaluation of GPT-4. J. Med. Ethics 50, 90–96. doi: 10.1136/jme-2023-109549 37945336

[B5] Carnat I. (2024). Human, all too human: accounting for automation bias in generative large language models. Internat. Data Priv. Law 14, 299–314. doi: 10.1093/idpl/ipae018

[B6] Chai Y. Xie H. Qin J. S. (2026). Text data augmentation for large language models: a comprehensive survey of methods, challenges, and opportunities. Artif. Intell. Rev. 59:35. doi: 10.1007/s10462-025-11405-5

[B7] Christiano P. F. Leike J. Brown T. Martic M. Legg S. Amodei D. . (2017). Deep reinforcement learning from human preferences. arXiv [Preprint]. arXiv:1706.03741. doi: 10.48550/arXiv.1706.03741

[B8] COPE Cases. Available online at: https://publicationethics.org/guidance?f%5B0%5D=type%3A44 (Accessed December 20 2025).

[B9] COPE Guidance. Available online at: https://publicationethics.org/guidance?f%5B0%5D=type%3A21 (Accessed December 20 2025).

[B10] COPE. Available online at: https://publicationethics.org/ (Accessed December 20 2025).

[B11] Foo J. Y. Wilson S. J. (2012). An analysis on the research ethics cases managed by the committee on publication ethics (COPE) between 1997 and 2010. Sci. Eng. Ethics 18, 621–631. doi: 10.1007/s11948-011-9273-3 21528428

[B12] Gallifant J. Afshar M. Ameen S. Aphinyanaphongs Y. Chen S. Cacciamani G. . (2025). The TRIPOD-LLM reporting guideline for studies using large language models. Nat. Med. 31, 60–69. doi: 10.1038/s41591-024-03425-5 39779929 PMC12104976

[B13] Huang H. Li Y. Jiang B. Liu L. Jiang B. Sun R. . (2024). Position: on-premises LLM deployment demands a middle path: preserving privacy without sacrificing model confidentiality. arXiv [Preprint] arXiv:2410.11182. doi: 10.18653/v1/2025.emnlp-main.420

[B14] Lane T. (2018). Journal publication ethics and implications for life science researchers: a COPE perspective. Emerg. Topics Life Sci. 2, 763–767. doi: 10.1042/ETLS20180164 33530669

[B15] Lee J. H. Shin J. (2024). How to optimize prompting for large language models in clinical research. Korean J. Radiol. 25, 869–873. doi: 10.3348/kjr.2024.0695 39344543 PMC11444847

[B16] Maity S. Saikia M. J. (2025). Large language models in healthcare and medical applications: a review. Bioengineering (Basel) 12:631. doi: 10.3390/bioengineering12060631 40564447 PMC12189880

[B17] Mann S. P. Seah J. J. Latham S. Savulescu J. Aboy M. Earp B. D. . (2025). Chat-IRB? How application-specific language models can enhance research ethics review. J. Med. Ethics Jme:110845. doi: 10.1136/jme-2025-110845 40764013

[B18] Protocol for LLMs Assessing COPE Queries. Available online at: https://osf.io/vumcp (Accessed December 20 2025).

[B19] Soffer S. Sorin V. Nadkarni G. N. Klang E. (2025). Pitfalls of large language models in medical ethics reasoning. NPJ Digit. Med. 8:461. doi: 10.1038/s41746-025-01792-y 40696098 PMC12284149

[B20] Sridharan K. Sivaramakrishnan G. (2024). Assessing the decision-making capabilities of artificial intelligence platforms as institutional review board members. J. Empir. Res. Human Res. Ethics 19, 83–91. doi: 10.1177/15562646241263200 38887060

[B21] Sridharan K. Sivaramakrishnan G. (2025). Leveraging artificial intelligence to detect ethical concerns in medical research: a case study. J. Med. Ethics 51, 126–134. doi: 10.1136/jme-2023-109767 38408853

[B22] Tarkang E. E. Kweku M. Zotor F. B. (2017). Publication practices and responsible authorship: a review article. J. Public Health Afr. 8:723. doi: 10.4081/jphia.2017.723 28748064 PMC5510206

[B23] Wager E. (2012). The committee on publication ethics (COPE): objectives and achievements 1997–2012. La Presse Médicale 41, 861–866. doi: 10.1016/j.lpm.2012.02.049 22727914

[B24] Wallat J. Jatowt A. Anand A. (2024). “Temporal blind spots in large language models,” in Proceedings of the 17th ACM International Conference on Web Search and Data Mining (pp. 683–692). doi: 10.1145/3616855.3635818

[B25] Zhang S. Dong L. Li X. Zhang S. Sun X. Wang S. . (2023). Instruction tuning for large language models: a survey. ACM Comp. Surveys 58, 1–36. doi: 10.1145/377741

[B26] Zhang Y. Li S. Qian C. Liu J. Yu P. Han C. . (2025). Law of knowledge overshadowing: towards understanding, predicting, and preventing llm hallucination. arXiv [Preprint]. arXiv:2502.16143. doi: 10.18653/v1/2025.fever-1.10

