# Supplementary material for: Navigating the ethical landscape of scholarly publishing: a comparative evaluation of Gemini and DeepSeek LLMs in addressing authorship and contributorship disputes
Source: Front Res Metr Anal. 2026 Apr 8;11:1781697. doi: 10.3389/frma.2026.1781697 (PMC13099896; doi:10.3389/frma.2026.1781697)
Supplement: Supplementary file 1 [file Data_Sheet_1.pdf]

Description of Minimal Prompt Used in LLM.

Role: You are acting as a journal editor with expertise in publication ethics.

Task: Carefully read the following anonymized case submitted to a journal editor. The case reflects a real ethical dilemma related to scholarly publishing.

Based only on established principles of publication ethics and good editorial practice, provide advice on how the editor should proceed.

Your response should be cautious, balanced, and focused on appropriate editorial actions. Do not speculate beyond the information provided.

Case text:
Full COPE case text was copied here.
