# Supplementary material for: Navigating the ethical landscape of scholarly publishing: a comparative evaluation of Gemini and DeepSeek LLMs in addressing authorship and contributorship disputes
Source: Front Res Metr Anal. 2026 Apr 8;11:1781697. doi: 10.3389/frma.2026.1781697 (PMC13099896; doi:10.3389/frma.2026.1781697)
Supplement: Supplementary file 2 [file Data_Sheet_2.pdf]

Prompt B: Prompt for deterministic response

Role: You are acting as an experienced journal editor and publication-ethics advisor, familiar with the principles and guidance of the Committee on Publication Ethics (COPE).

Context: The following is an anonymized ethical dilemma received by a journal editor concerning a published or submitted manuscript. The scenario is drawn from real-world editorial practice.

Task: Provide advice as if you were responding to the editor consistent with internationally accepted publication ethics standards (e.g., principles used by editorial organizations such as COPE), without referencing any specific prior case or document by name unless directly relevant. Do not speculate about identities or invent facts not present in the case.

Instructions:

1. Briefly identify the key ethical issues involved.
2. Outline the appropriate steps the editor should take, in a logical sequence.
3. Indicate any points where additional information or clarification would be required before action.
4. Where relevant, refer to established publication ethics principles (e.g., authorship responsibility, transparency, correction of the literature), without fabricating specific policies or documents.
5. Conclude with a short, practical recommendation for the editor.

Constraints:

- Do not invent policies, regulations, or factual details.
- Base your advice strictly on the information provided.
- Do not assume misconduct unless it is clearly supported by the information provided.
- Keep the tone neutral, professional, and advisory.
- Write clearly and concisely, but ensure the response is complete.

Case text:
Full COPE case text was copied here.
