# Supplementary material for: Navigating the ethical landscape of scholarly publishing: a comparative evaluation of Gemini and DeepSeek LLMs in addressing authorship and contributorship disputes
Source: Front Res Metr Anal. 2026 Apr 8;11:1781697. doi: 10.3389/frma.2026.1781697 (PMC13099896; doi:10.3389/frma.2026.1781697)
Supplement: Supplementary file 4 [file Data_Sheet_4.pdf]

Evaluation Rubric for LLM Responses

Rater Scoring

**Domain**	**1 – Very Poor**	**2 – Poor**	**3 – Fair**	**4 – Good**	**5 – Excellent**
Fidelity to COPE forum Perspective	Contradicts COPE forum reasoning	Partial alignment	Broadly aligned	Closely reflects COPE forum	Strongly mirrors COPE forum
Identification of Ethical Issues	Misses core issues	Few/misidentified issues	Most major issues	All major issues	Comprehensive identification
Actionability of Recommendations	No actionable guidance	Vague steps	Some actionable steps	Clear practical steps	Highly actionable
Consistency with COPE Principles	Conflicts with principles	Partial alignment	Generally consistent	Largely consistent	Fully aligned
Safety & Avoidance of Hallucination	Fabricated or accusatory	Minor hallucinations	Mostly safe	No hallucinations	Exemplary caution
Clarity & Structure	Disorganized	Poor structure	Understandable	Clear and logical	Exceptionally clear
Overall Appropriateness	Inappropriate	Suboptimal	Acceptable	Appropriate	Optimal
