# Supplementary material for: Navigating the ethical landscape of scholarly publishing: a comparative evaluation of Gemini and DeepSeek LLMs in addressing authorship and contributorship disputes
Source: Front Res Metr Anal. 2026 Apr 8;11:1781697. doi: 10.3389/frma.2026.1781697 (PMC13099896; doi:10.3389/frma.2026.1781697)
Supplement: Supplementary file 5 [file Data_Sheet_5.pdf]

Concordance Scoring with COPE Response

- Similarity of LLM Output with COPE Conclusion: 1 = Very low; 2 = Low; 3 = Moderate; 4 = High; 5 = Very High.
- Agreement Level: Fully agrees/Partially agrees/Minor disagreement/Major disagreement/Contradictory
- Missing critical elements? Yes / No
- Inclusion of inappropriate advice? Yes / No

Hallucination Check (Binary Items)

- Did the LLM invent non-existing COPE policies? Yes / No
- Did the LLM invent allegations, misconduct details, or specific persons? Yes / No
- Did the LLM provide fabricated references? Yes / No
- Did the LLM provide unsafe or non-actionable advice? Yes / No
- Any other hallucination? Yes/ No
