# Supplementary material for: Navigating the ethical landscape of scholarly publishing: a comparative evaluation of Gemini and DeepSeek LLMs in addressing authorship and contributorship disputes
Source: Front Res Metr Anal. 2026 Apr 8;11:1781697. doi: 10.3389/frma.2026.1781697 (PMC13099896; doi:10.3389/frma.2026.1781697)
Supplement: Supplementary file 6 [file Data_Sheet_6.pdf]

Original response from the individual LLMs can be accessed through the following links:

Google Gemini 2.5 Flash response: https://gemini.google.com/share/c3bb6ba95774

DeepSeek responses: https://chat.deepseek.com/share/8w5ci1klnc8yea0591

https://chat.deepseek.com/share/g1qk4ypeouscxhiiqp

https://chat.deepseek.com/share/2uxgyj49mzl4iv7dg5
