# Supplementary material for: Navigating the ethical landscape of scholarly publishing: a comparative evaluation of Gemini and DeepSeek LLMs in addressing authorship and contributorship disputes
Source: Front Res Metr Anal. 2026 Apr 8;11:1781697. doi: 10.3389/frma.2026.1781697 (PMC13099896; doi:10.3389/frma.2026.1781697)
Supplement: Supplementary file 7 [file Data_Sheet_7.pdf]

Detailed Assessment of LLM Response for the Cases included in Authorship and Contributorship Domain.

Case number	Prompt type	Google Gemini 2.5 Flash	DeepSeek-V3.2
20-07	Minimal	• Did not state that authorship issues should not be the sole purpose for retraction (Stated in COPE response).<br>• Did not recommend the Editor to check for breach of copyright infringement/legal issues by the AOR (Stated in COPE response).<br>• Did not state that journals should update their instructions to authors mentioning that the manuscripts will not be retracted solely based on authorship dispute (Stated in COPE response).<br>• Clearly answered the query on resubmission and potential sanctions to be considered. *<br>• Clearly enumerates the steps to be taken to be addressed to the authors. *<br>• Explicitly states that the AOR has done scientific misconduct.	• Did not state that authorship issues should not be the sole purpose for retraction (Stated in COPE response).<br>• Did not recommend the Editor to check for breach of copyright infringement/legal issues by the AOR (Stated in COPE response).<br>• Did not state that journals should update their instructions to authors mentioning that the manuscripts will not be retracted solely based on authorship dispute (Stated in COPE response).<br>• Clearly answered the query on resubmission. *<br>• Did not explicitly state that the AOR has done scientific misconduct.<br>• Clearly enumerates the steps to be taken to be addressed to the authors. *
	Deterministic	• Provides a detailed step-by-step sequence for formal documentation and final confirmation from all authors for retraction.<br>• Explicitly states that authorship disputes are often not the reason for retraction.<br>• Suggests obtaining co-authors' stance on retraction. *<br>• Did not recommend the Editor to check for breach of copyright infringement/legal issues by the AOR (Stated in COPE response).<br>• Did not state that journals should update their instructions to authors mentioning that the	• Did not state that authorship issues should not be the sole purpose for retraction (Stated in COPE response).<br>• Did not recommend the Editor to check for breach of copyright infringement/legal issues by the AOR (Stated in COPE response).<br>• Did not state that journals should update their instructions to authors mentioning that the manuscripts will not be retracted solely based on authorship dispute (Stated in COPE response).<br>• Did not explicitly state that the AOR has done scientific misconduct.

		manuscripts will not be retracted solely based on authorship dispute (Stated in COPE response).	• Suggests verifying the co-authors' position on the retraction. *
	Stochastic	• Suggests obtaining co-authors' stance on retraction. *<br>• States that the editor's decision on retraction is justified as the AOR persists in retracting the article.<br>• Mentions that AOR's stand dictates outcome.<br>• Did not state that journals should update their instructions to authors mentioning that the manuscripts will not be retracted solely based on authorship dispute (Stated in COPE response).	• Recommends stating a letter indicating that retraction notice will be published if an unanimously written agreement on author order is not received. *<br>• For future reference, it recommends sending an automatic mail to co-authors to confirm their authorship. *<br>• Did not state that journals should update their instructions to authors mentioning that the manuscripts will not be retracted solely based on authorship dispute (Stated in COPE response).
20-08	Minimal	• States that the reason for re-rejection should appropriately based on the original scope/novelty/technical concerns rather than stating "borderline bullying". *<br>• Insists that the journal should state that it will not encourage any further correspondence regarding the editorial decision following re-rejection. *<br>• Suggests documenting to corresponding author, copying the senior author that using social media polls and threatening to influence editorial decisions constitute unprofessional and unacceptable behavior. *<br>• Suggests communicating that the future submission from this author will be subjected to stricter scrutiny if similar behavior is repeated. *<br>• Suggests incorporating structured recommendations for having appeal policy, desk rejection communications, and code of conduct for authors and reviewers. *	• States that the reason for re-rejection should appropriately based on the original scope/novelty/technical concerns rather than stating "borderline bullying". *<br>• Suggests structured appeal response as follows: "Your appeal has been reviewed by senior editors. The original decision stands as a final editorial judgment based on the manuscript's fit with the journal's scope and priorities. The journal's policy is that editorial decisions on suitability for peer review are final and not subject to further debate" *<br>• Suggests having a publicly displayed appeal policy mentioning that decisions on desk rejection are typically final and not subject to appeal or outline very limited grounds for appeal (such as procedural error and non-substantive disagreement.

		• Recommends following up with the senior author on the internal action taken for completing the loop. *<br>• Did not provide the possible author's perspective where a possibility of difficult personality with misdirected enthusiasm who wish to learn from the journal editor (Stated in COPE response).	• Suggests developing a standardized template response for desk rejections and appeals that are polite, firm, and consistent for reducing ambiguity and emotional reactivity. *<br>• Suggests providing training on de-escalation strategy for the editors. *<br>• Did not provide the possible author's perspective where a possibility of difficult personality with misdirected enthusiasm who wish to learn from the journal editor (Stated in COPE response).
	Deterministic	• Suggests communicating desk rejections with brief explanation for ensuring transparency of the process. *<br>• Suggests maintaining a comprehensive internal record of the entire exchange for future reference. *<br>• Recommends seeking clarification from the senior author on the details of their internal action. *<br>• Suggests citing the existing code of conduct for authors in the final communication. *	• Suggests formalizing the initial appeal process where the handling editor should have responded politely providing a point-by-point rebuttal, citing the journal's policy on the finality of editorial pre-screening decisions. *<br>• Suggests that the section head's response to the author stating that the editorial deliberations are confidential and not shareable, and the editorial decision is final, and the correspondence is considered closed. *<br>• Recommends carrying out an internal check on the social media poll to ensure that it does not misrepresent journal/editors. *
	Stochastic	• Suggests that editor should consult the legal department to ensure that the journal is not subject to freedom of information laws. *<br>• Suggests informing the unprofessional conduct of the author to all coauthors. *	• Suggests that editor must have mentioned at the time of first appeal that editorial decisions are based on the expert judgment of its editors regarding scope and priority and cannot be informed by public polling or comparisons to other published work. *
20-10	Minimal	• Suggests that the author's personal career is external to the scholarly communication system.	• Suggests not removing the article from the Crossref database.

		• Suggests that retraction should be carried out only in cases of major error or scientific misconduct and retraction notice should remain permanent.<br>• Recommends the publisher to reinstate the article if it is retracted. *<br>• Suggests that if the author resubmits the article to another journal, ethical rules regarding the redundant publication must be adhered to where the full details (including DOI number) of the published article and permission from the previous publisher must be disclosed in the cover letter. *	• The author's reasons for request are not related to the validity of the article.<br>• Suggests the possibility of retraction could be unfair to other authors and could damage the trust in the publishing system. *<br>• Recommends that the author should engage in discussion with the institution explaining the circumstances and not seek alteration in the publication record.<br>• Provides suggestions to the author for considering to significantly expand or transform study with citation of the original work to a new journal. *
	Deterministic	• Recommends the publisher to reinstate the article if it is retracted. *<br>• Recommends the editor to issue an editorial note on reinstating the retracted article mentioning the DOI of the original article, reason for retraction and reinstatement, and the reinstatement was done for upholding the integrity of publication ethics. *<br>• Suggests reactivating the DOI, if it is retired for this article. *	• Outlines the key information to be communicated to the author in declining the request for retraction. *<br>• Recommends the Crossref® to internally clarify whether the publisher has a stated policy on article withdrawal for reasons other than error or misconduct that might help Crossref® understand the publishing practices that can inform its own policy stance. *
	Stochastic	• Explicitly states that the publisher has committed a serious ethical error in retracting the article. *<br>• Recommends the editor to issue an editorial note on reinstating the retracted article mentioning the DOI of the original article, reason for retraction and reinstatement, and the reinstatement was done for upholding the integrity of publication ethics. *	• Identifies the issue of impact of inflexible institutional research assessment policies that should not override publication ethics. *<br>• States that if the publisher permits withdrawal for non-ethical reasons, it creates a disconnect that Crossref's policy may need to address explicitly. *
20-11	Minimal	• Recommend sending one final letter to the authors using all the available email addresses informing them of the urgency and consequences of not responding.	• Recommend sending one final letter to the authors using all the available email addresses informing them of the urgency and consequences of not responding.

		• Mentions that under rare circumstances, if the manuscript addresses a matter of immediate and significant public health or safety, and the only missing element is minor copyediting approval, the editor might consider publishing with an Editorial note stating that the authors were unavailable for final proof review. However, it also identifies that this approach is highly risk and generally not recommended. *<br>• Did not recommend considering the signature of journal publishing agreement that could possibly imply legal and copyright violations (stated in COPE response).	• States that if the authors contacted the journal later, the journal should be willing to discuss the possibility of resubmission, but the manuscript should be subjected to a new editorial assessment. *<br>• Did not recommend considering the signature of journal publishing agreement that could possibly imply legal and copyright violations (stated in COPE response).
	Deterministic	• Suggests emphasizing to the editor that to protect the author's interest, the author must be informed that they retain the right to submit the manuscript elsewhere after obtaining formal approval from the journal. *<br>• Did not recommend considering the signature of journal publishing agreement that could possibly imply legal and copyright violations (stated in COPE response).	• Clarifies whether the journal has a policy related to unresponsive authors and if not suggests creating one. *<br>• Did not recommend considering the signature of journal publishing agreement that could possibly imply legal and copyright violations (stated in COPE response).
	Stochastic	• Recommends the journal to consider flagging the corresponding author's file in its internal system identifying the history of non-cooperation post-acceptance, so that editorial members will be aware of such instances during any of the future submissions. *<br>• Did not recommend considering the signature of journal publishing agreement that could possibly imply legal and copyright violations (stated in COPE response).	• Did not recommend considering the signature of journal publishing agreement that could possibly imply legal and copyright violations (stated in COPE response).

20-15	Minimal	• Suggests obtaining clarification from the institution about whether the investigation is complete and recommends obtaining the final investigation report. *<br>• Recommends the editor to consult with the legal team and the senior authors to determine whether the unacknowledged ideas and results undermine the originality, novelty, or core scientific findings in which case retraction notice must be issued and if not, notice of concern to be issued. *<br>• Details the content for the notice of concern. *<br>• Did not specify threatening the authors with retraction in case no agreement could be reached (stated in COPE response).<br>• Did not recommend the editor contacting the second institution (stated in COPE response).	• Details the content for the notice of concern. *<br>• Did not specify threatening the authors with retraction in case no agreement could be reached (stated in COPE response).<br>• Did not recommend the editor contacting the second institution (stated in COPE response).
	Deterministic	• Suggests obtaining clarification from the institution about whether the investigation is complete and recommends obtaining the final investigation report as well as the statement that the institution has exhausted all internal appeal routes. *<br>• Recommends the editor to determine whether the unacknowledged ideas and results undermine the originality, novelty, or core scientific findings in which case retraction notice must be issued and if not, notice of concern to be issued. *<br>• Did not specify threatening the authors with retraction in case no agreement could be reached (stated in COPE response).<br>• Did not recommend the editor contacting the second institution (stated in COPE response).	• Suggests the editor in obtaining further information on whether the investigation findings relate to this published paper, and whether the institution recommends any specific action to be taken (erratum/retraction) or allows the journal to decide on the specific course of action. *<br>• In cases of disputes between the authors and their institutions, the journal should act on the formal institutional findings. *<br>• Did not recommend the editor contacting the second institution (stated in COPE response).

	Stochastic	• Recommends the editor to determine whether the unacknowledged ideas and results undermine the originality, novelty, or core scientific findings in which case retraction notice must be issued and if not, notice of concern to be issued. *<br>• Did not specify threatening the authors with retraction in case no agreement could be reached (stated in COPE response).<br>• Did not recommend the editor contacting the second institution (stated in COPE response).	• Suggests obtaining clarification from the institution on whether they followed the standard formally established procedures for research misconduct inquiries. *<br>• Suggests that the expression of concern could be escalated to a retraction in cases of overwhelming public evidence. *<br>• Did not specify threatening the authors with retraction in case no agreement could be reached (stated in COPE response).<br>• Did not recommend the editor contacting the second institution (stated in COPE response).
20-17	Minimal	• Journal editor's primary mandate is to address research integrity and publication misconduct. It is beyond the core mandate to get involved in non-research related issues as is the case.<br>• The co-author, having met the authorship criteria according to ICMJE/CRediT criteria, carries accountability and removing their authorship absolves them of that public accountability. *<br>• Reclassifying the coauthor as a contributor after the agreement from all authors can be considered. *<br>• Provide a choice to the co-author for publishing "Author Disassociation Statement" alongside the article, indicating the co-author's desire to publicly disassociate from the lead author. *	• Suggests retaining the coauthor and not moving to contributor as it will contradict the CRediT role originally selected. *<br>• The coauthor removal can be considered following submission of a formal report from the institution on the findings that impact authorship. *
	Deterministic	• Journal editor's primary mandate is to address research integrity and publication misconduct. It is beyond the core mandate to get involved in non-research related issues as is the case.<br>• Explicitly states that removing an author whose contribution is acknowledged simply due to	• Specifies that there is no editorial mandate to clearly alter the publication record in this case. *<br>• Suggests that removal of coauthor is possible only if all authors, including the lead author, genuinely agree. *

		reputational damage caused by a co-author sets an undesirable precedent that fundamentally compromises the integrity of the author list. *<br>• Co-authors have the accountability as stated in the original contribution and removing the co-author absolutely absolves them of this public accountability.<br>• Suggests the editor to consult the legal counsel before publishing any notice. *<br>• Provide a choice to the co-author for publishing "Author Disassociation Statement" alongside the article, indicating the co-author's desire to publicly disassociate from the lead author. *<br>• Did not suggest reclassifying the coauthor as a contributor after the agreement from all authors can be considered unlike with the minimal prompt.	
	Stochastic	• Journal editor's primary mandate is to address research integrity and publication misconduct. It is beyond the core mandate to get involved in non-research related issues as is the case.<br>• Explicitly states that removing an author whose contribution is acknowledged simply due to reputational damage caused by a co-author sets an undesirable precedent that fundamentally compromises the integrity of the author list. *<br>• Did not suggest reclassifying the coauthor as a contributor after the agreement from all authors can be considered unlike with the minimal prompt.<br>• Suggests the editor to consult the legal counsel before publishing any notice. *	• States that by removing the co-author, the editor respects author autonomy but introduce significant ethical complexity. *<br>• Suggests that removal of co-author is possible following a formal, unanimous request for removal from all original authors. *<br>• Suggests that concerns about a co-author's professional conduct are best addressed within their own institution or professional networks, not through alteration of the publication record. *

		• Provide a choice to the co-author for publishing "Author Disassociation Statement" alongside the article, indicating the co-author's desire to publicly disassociate from the lead author. *	
20-18	Minimal	• Suggests informing the authors that the journal will no longer mediate this dispute, nor will it accept further phone calls or personal communication regarding the authorship claim. *<br>• Refer the dispute issue to the authors' institutions and request formal investigations to be carried out.<br>• Recommends the editor for obtaining tangible evidence for supporting the claim of substantive contribution to the manuscript drafting (e.g., emails, dated data files, tracked changes) before contacting the university. *<br>• Recommends the editor to keep status quo for the disputed article until the institution sends a formal documented finding of their investigation. *	• Details the elements the editor must brief the institution of the lead author about the authorship dispute. *<br>• Suggests adding a temporary editorial note that can be removed or changed to corrigendum or expression of concern following the results of institutional investigation. *<br>• If the institution declines to investigate, it suggests the editor should investigate the dispute and as there is no written agreement, the case should be closed without adding the complainant author and should be intimated. *<br>• Explicitly states that the phone calls are harassing and unprofessional and the authors should be informed that the only channel of communication is through official email addresses. *
	Deterministic	• States that the journal office is not equipped to handle authorship dispute cases, particularly in the absence of written agreement.<br>• Suggests informing the authors that the journal's decision on altering the published author list will be based solely on the formal, documented findings of the institutional investigation. *<br>• Recommends the editor to keep status quo for the disputed article until the institution sends a formal documented finding of their investigation. *<br>• Recommends the editor for obtaining tangible evidence for supporting the claim of substantive	• Insists on maintaining professional boundaries by informing the authors that phone calls will not be accepted as they hinder the fair evaluation process. *<br>• Details the content of the temporary editorial note for maintaining transparency as follows: "The journal has been alerted to a dispute regarding authorship contributions. This matter has been referred to the authors' institution for investigation." *<br>• If the institution declines to investigate, it suggests the editor should investigate the

		contribution to the manuscript drafting (e.g., emails, dated data files, tracked changes) before contacting the university in a short window. *	dispute and as there is no written agreement, the case should be closed without adding the complainant author and should be intimated. *<br>• Suggests fetching the institution details of the complainant author and if it different, the editor should be contacting both the institutions. *<br>• Suggests the editor to create a policy on resolving author's disputes, if there is none. *
	Stochastic	• Suggests the editor that the harassment by phone calls should be stopped. *<br>• Suggests the editor to provide a final, short deadline for both authors to sign a Joint Authorship Agreement that specifies the complaining author's role before referral to the institution. *	• Provides explicit details on the contents mentioned in the communication letters to be provided to both the authors and their institutions. *<br>• Details the content of the temporary editorial note for maintaining transparency as follows: "This article has been subject to an authorship contribution dispute. The matter has been referred to the authors' institution(s) for investigation. Further editorial action will be informed by the outcome of that process." *<br>• Suggests obtaining more clarification about the specific statistical analyses performed, their intellectual input into the interpretation, and whether this work is directly identifiable in the final paper. *<br>• Recommends obtaining the collective position regarding the sufficiency of the complaining individual's contribution. *<br>•
20-20	Minimal	• The authors harassment emails to the journal B tantamount to professional misconduct. *<br>• Research from non-academic institutions is not a problem provided veracity is confirmed.	• Suggests carrying out an internal audit focusing on ethics approval, data availability, internal consistency and similarities to the manuscript rejected by another journal. *

		• States that the rejection of the existing manuscript by journal A is appropriate. *<br>• Suggests sending a registered letter or email to provide formal, verifiable documentation on their affiliations that matches the ones mentioned in the manuscript. *<br>• Consult with legal counsel. *<br>• Suggests expressing the note of concern for both published articles in case of no response from the authors. *<br>• Suggests banning future submissions from the author if the affiliation cannot be verified. *<br>• Did not state that it is not correct for providing unsolicited information to a journal editor (stated in the COPE forum response).	• Suggests sending email to provide formal, verifiable documentation on their affiliations that matches the ones mentioned in the manuscript. *<br>• In case of verified authors' affiliation that is convincing to the editor, the manuscript should not be rejected and shall continue the standard editorial and peer review process as per the journal's instructions. *<br>• Suggests expressing the note of concern for both published articles in case of no response from the authors. *<br>• Suggests informing journal B in case their affiliations cannot be verified.<br>• Did not state that it is not correct for providing unsolicited information to a journal editor (stated in the COPE forum response).
	Deterministic	• The authors harassment emails to the journal B tantamount to professional misconduct. *<br>• Research from non-academic institutions is not a problem provided veracity is confirmed.<br>• States that the rejection of the existing manuscript by journal A was sound. *<br>• Suggests sending a registered letter or email to provide formal, verifiable documentation on their affiliations that matches the ones mentioned in the manuscript. *<br>• Consult with legal counsel. *<br>• Suggests expressing the note of concern for both published articles in case of no response from the authors. *<br>• Suggests banning future submissions from the author if the affiliation cannot be verified. *	• Suggests carrying out an internal audit focusing on ethics approval, data availability, internal consistency and similarities to the manuscript rejected by another journal. *<br>• Suggests sending email to provide formal, verifiable documentation on their affiliations that matches the ones mentioned in the manuscript. *<br>• In case of verified authors' affiliation that is convincing to the editor, the manuscript should not be rejected and shall continue the standard editorial and peer review process as per the journal's instructions. *<br>• Suggests expressing the note of concern for both published articles in case of no response from the authors. *

		• Did not state that it is not correct for providing unsolicited information to a journal editor (stated in the COPE forum response).	• Suggests informing journal B in case their affiliations cannot be verified.<br>• Suggests Journal A editor to fetch more details from Journal B for any additional documentary evidence they gathered (e.g., screenshots of registry searches, copies of the harassing emails with headers) to aid in the verification process. *<br>• Did not state that it is not correct for providing unsolicited information to a journal editor (stated in the COPE forum response).
	Stochastic	• The authors harassment emails to the journal B tantamount to professional misconduct. *<br>• Research from non-academic institutions is not a problem provided veracity is confirmed.<br>• States that the rejection of the existing manuscript by journal A was sound. *<br>• Suggests sending a registered letter or email to provide formal, verifiable documentation on their affiliations that matches the ones mentioned in the manuscript. *<br>• Consult with legal counsel. *<br>• Suggests expressing the note of concern for both published articles in case of no response from the authors. *<br>• Suggests banning future submissions from the author if the affiliation cannot be verified. *<br>• Did not state that it is not correct for providing unsolicited information to a journal editor (stated in the COPE forum response).	• Suggests carrying out an internal audit focusing on ethics approval, data availability, internal consistency and similarities to the manuscript rejected by another journal. *<br>• Suggests sending email to provide formal, verifiable documentation on their affiliations that matches the ones mentioned in the manuscript. *<br>• In case of verified authors' affiliation that is convincing to the editor, the manuscript should not be rejected and shall continue the standard editorial and peer review process as per the journal's instructions. *<br>• Suggests expressing the note of concern for both published articles in case of no response from the authors. *<br>• Suggests informing journal B in case their affiliations cannot be verified.<br>• Suggests Journal A editor to fetch more details from Journal B for any additional documentary evidence they gathered (e.g., screenshots of registry searches, copies of the harassing emails with headers) to aid in the verification process. *

			• Did not state that it is not correct for providing unsolicited information to a journal editor (stated in the COPE forum response).
20-23	Minimal	• Explicitly states that indefinite holding of the paper is not good editorial practice.<br>• Suggests rejecting the manuscript.<br>• Advises the journal to avoid conducting a parallel, time-consuming investigation into the external data source if a high-profile, formal investigation is already underway by other major publishers/institutions. *<br>• Suggests actively monitoring the external investigation for their final reports, following which, if it is concerning, the author group should be flagged for internal purposes. *<br>• Did not provide the option of communicating to the author to wait until the investigation is completed and inform the editor on the results of the investigation, following which a decision can be rendered (stated in the COPE forum response).<br>• Did not suggest that the journal can also fetch the data and seek advice from the experts on the veracity (stated in the COPE forum response).<br>• Did not suggest verifying for the possibility of false declaration at the submission time should also be explored (stated in the COPE forum response).	• Suggests rejecting the manuscript.<br>• The details of rejecting this manuscript along with the underlying reasons can be shared with other journals, if they are in the same consortium. *<br>• The editor must monitor the situation continuously.<br>• Did not provide the option of communicating to the author to wait until the investigation is completed and inform the editor on the results of the investigation, following which a decision can be rendered (stated in the COPE forum response).<br>• Did not suggest that the journal can also fetch the data and seek advice from the experts on the veracity (stated in the COPE forum response).<br>• Did not suggest verifying for the possibility of false declaration at the submission time should also be explored (stated in the COPE forum response).
	Deterministic	• Suggests internal flagging for any potential submission by the author group in future. *<br>• Explicitly states that indefinite holding of the paper is not good editorial practice.<br>• Suggests rejecting the manuscript.	• Specifies the exact details to be stated to the author while rejecting the manuscript as follows: “"The journal has become aware of significant concerns regarding the integrity and provenance of the data source central to this study. These concerns, which are the subject of ongoing

		• Did not provide the option of communicating to the author to wait until the investigation is completed and inform the editor on the results of the investigation, following which a decision can be rendered (stated in the COPE forum response).<br>• Did not suggest that the journal can also fetch the data and seek advice from the experts on the veracity (stated in the COPE forum response).<br>• Did not suggest verifying for the possibility of false declaration at the submission time should also be explored (stated in the COPE forum response).	investigations related to other publications using the same data, prevent us from proceeding with publication at this time."<br>• Did not provide the option of communicating to the author to wait until the investigation is completed and inform the editor on the results of the investigation, following which a decision can be rendered (stated in the COPE forum response).<br>• Did not suggest that the journal can also fetch the data and seek advice from the experts on the veracity (stated in the COPE forum response).<br>• Did not suggest verifying for the possibility of false declaration at the submission time should also be explored (stated in the COPE forum response).
	Stochastic	• Suggests internal flagging for any potential submission by the author group in future. *<br>• Explicitly states that indefinite holding of the paper is not good editorial practice.<br>• Suggests rejecting the manuscript.<br>• Did not provide the option of communicating to the author to wait until the investigation is completed and inform the editor on the results of the investigation, following which a decision can be rendered (stated in the COPE forum response).<br>• Did not suggest that the journal can also fetch the data and seek advice from the experts on the veracity (stated in the COPE forum response).<br>• Did not suggest verifying for the possibility of false declaration at the submission time should also be explored (stated in the COPE forum response).	• Provides more details to be mentioned in the communication when rejecting the manuscript. *<br>• Suggests providing an opportunity for authors to revert if authors provide compelling evidence following the completion of external investigation. *<br>• Did not provide the option of communicating to the author to wait until the investigation is completed and inform the editor on the results of the investigation, following which a decision can be rendered (stated in the COPE forum response).<br>• Did not suggest that the journal can also fetch the data and seek advice from the experts on the veracity (stated in the COPE forum response).<br>• Did not suggest verifying for the possibility of false declaration at the submission time should

			also be explored (stated in the COPE forum response).
20-25	Minimal	• Identifies that the journal has a policy where authors can be added before the acceptance of the manuscripts and so any changes will violate this policy. *<br>• States that it is generally considered highly unethical to request a significant change in authorship at this extremely late stage. *<br>• States that this practice arises a suspicion of gift authorship. *<br>• States that the two requests related to authorship changes are conflicting further raising integrity concerns. *<br>• Suggests that the specified contribution by the new author justifies acknowledgement rather than authorship.<br>• Suggests that the editor has the sole responsibility to reject a manuscript based on ethical concerns. *<br>• Suggests the editor to obtain the details of contribution by the new author according to the ICMJE criteria and if it does not meet the criteria, reject. If the suggestions meet the criteria, then reject it based on the journal's late-stage policy. *	• States that the reasons claimed for authorship in this case are primarily administrative/technical.<br>• Identifies that the journal has a policy where authors can be added before the acceptance of the manuscripts and so any changes will violate this policy. *<br>• States that it is generally considered unethical to request a significant change in authorship at this extremely late stage. *<br>• States that this practice arises a suspicion of gift authorship. *<br>• Suggests that the editor has the sole responsibility to reject a manuscript based on ethical concerns. *<br>• Recommends the editor should reject the manuscript and in case of authors disagreement, the editor can withdraw the acceptance of the manuscript. *
	Deterministic	• Identifies that the journal has a policy where authors can be added before the acceptance of the manuscripts and so any changes will violate this policy. *<br>• States that it is generally considered highly unethical to request a significant change in authorship at this extremely late stage. *<br>• Suggests that the editor has the sole responsibility to reject a manuscript based on ethical concerns. *	• States that peer review and revision were conducted with the understanding of a specific author team and so change in authorship disrupts the integrity of editorial process. *<br>• States that the reasons claimed for authorship in this case are primarily logistic and do not warrant authorship.<br>• Suggests the editor to communicate the authors that if the authors accept with the existing

		• Suggests the editor to obtain the details of contribution by the new author according to the ICMJE criteria and if it does not meet the criteria, reject. If the suggestions meet the criteria, then reject it based on the journal's late-stage policy. *<br>• States that the two requests related to authorship changes are conflicting further raising integrity concerns. *	names, proceed with the publication and if they insist adding the new author, it should be rejected. *
	Stochastic	• Identifies that the journal has a policy where authors can be added before the acceptance of the manuscripts and so any changes will violate this policy. *<br>• States that it is generally considered highly unethical to request a significant change in authorship at this extremely late stage. *<br>• Suggests that the editor has the sole responsibility to reject a manuscript based on ethical concerns. *<br>• Suggests the editor to obtain the details of contribution by the new author according to the ICMJE criteria and if it does not meet the criteria, reject. If the suggestions meet the criteria, then reject it based on the journal's late-stage policy. *<br>• States that the two requests related to authorship changes are conflicting further raising integrity concerns. *	• States that peer review and revision were conducted with the understanding of a specific author team and so change in authorship disrupts the integrity of editorial process. *<br>• States that the reasons claimed for authorship in this case are primarily logistic and do not warrant authorship.<br>• Suggests the editor to communicate the authors that if the authors accept with the existing names, proceed with the publication and if they insist adding the new author, it should be rejected. *
20-29	Minimal	• States that publishing the same manuscript (consensus guidelines) in two different journals simultaneously constitute redundant publication, wasting the editorial and peer review resources. *<br>• Mentions that publishing consensus guidelines with different author orders introduce ambiguity for readers, citation analysis, and systematic reviews.	• Suggests publishing the manuscript in one society journal, with a notice or republication in the other as a shorter notice, editorial, or summary that explicitly references and links to the full guidelines published in the primary journal. This must be done with full transparency, permission from the primary journal's publisher, and clear attribution.

		• Insists on publishing the manuscript in only one journal. *<br>• Suggests that the only ethically permissible route is for one journal to publish the primary version, and the other journal may consider publishing it as a secondary version that is verbatim identical (including author order) and carries a prominent notice stating it is a reprint of the article first published in the other journal. *<br>• Did not state the authorship can be given as a collective group with the individuals being listed separately in variant order across journals (stated in the COPE forum response).<br>• Did not state that if joint publication with the same order is not acceptable, one journal could offer to take the lead in publishing it as a full document, while the other could publish the executive summary (stated in the COPE forum response).	• Suggests publishing the manuscript in a third, neutral single journal. *<br>• Insists on publishing the manuscript in only one journal. *<br>• States that publishing the consensus guidelines in two different journals simultaneously constitute redundant publication, wasting the editorial and peer review resources. *<br>• Did not state the authorship can be given as a collective group with the individuals being listed separately in variant order across journals (stated in the COPE forum response).<br>• Did not state that if joint publication with the same order is not acceptable, one journal could offer to take the lead in publishing it as a full document, while the other could publish the executive summary (stated in the COPE forum response).
	Deterministic	• Lists the exceptional situations where the same work (or one with substantial overlap) can be published in more than one journal, primarily these are the official translation/republication of core documents (like guidelines) with full transparency.<br>• Mentions that publishing consensus guidelines with different author orders introduce ambiguity for readers, citation analysis, and systematic reviews.<br>• Insists on publishing the manuscript in only one journal. *<br>• Suggests that the only ethically permissible route is for one journal to publish the primary version,	• Suggests obtaining clarification from the corresponding author that their intention is to publish two identical full manuscripts with different author orders. *<br>• Suggests publishing the manuscript in one society journal, with a notice or republication in the other as a shorter notice, editorial, or summary that explicitly references and links to the full guidelines published in the primary journal. This must be done with full transparency, permission from the primary journal's publisher, and clear attribution.<br>• Suggests publishing the manuscript in a third, neutral single journal. *

		and the other journal may consider publishing it as a secondary version that is verbatim identical (including author order) and carries a prominent notice stating it is a reprint of the article first published in the other journal. *<br>• Did not state the authorship can be given as a collective group with the individuals being listed separately in variant order across journals (stated in the COPE forum response).	• Insists on publishing the manuscript in only one journal. *
	Stochastic	• Lists the exceptional situations where the same work (or one with substantial overlap) can be published in more than one journal, primarily these are the official translation/republication of core documents (like guidelines) with full transparency.<br>• Mentions that publishing consensus guidelines with different author orders introduce ambiguity for readers, citation analysis, and systematic reviews.<br>• Insists on publishing the manuscript in only one journal. *<br>• Suggests that the only ethically permissible route is for one journal to publish the primary version, and the other journal may consider publishing it as a secondary version that is verbatim identical (including author order) and carries a prominent notice stating it is a reprint of the article first published in the other journal. *<br>• Did not state the authorship can be given as a collective group with the individuals being listed separately in variant order across journals (stated in the COPE forum response).	• Suggests obtaining clarification from the corresponding author that their intention is to publish two identical full manuscripts with different author orders. *<br>• Suggests publishing the manuscript in one society journal, with a notice or republication in the other as a shorter notice, editorial, or summary that explicitly references and links to the full guidelines published in the primary journal. This must be done with full transparency, permission from the primary journal's publisher, and clear attribution.<br>• Suggests publishing the manuscript in a third, neutral single journal. *<br>• Insists on publishing the manuscript in only one journal. *

20-30	Minimal	• Agrees that the journal has taken appropriate action.<br>• Clearly specifies the step-by-step actions. *<br>• Suggests providing one final opportunity for researchers for professionally editing the comment as suggested by the reviewer, and if the researcher fails to abide by this, the journal should consider the case closed and informing the parties concerned. *<br>• Suggests pausing any decision on issuing retraction/notice until the institutional investigation is completed.	• Agrees that the journal has taken appropriate action.<br>• Suggests providing one final opportunity for researchers for professionally editing the comment as suggested by the reviewer, and if the researcher fails to abide by this, the journal should consider the case closed and informing the parties concerned. *<br>• Did not state that in case the journal wishes to follow up on the veto power of the researcher, they must ask for appropriate proof (stated in the COPE forum response).
	Deterministic	• Agrees that the journal has taken appropriate action.<br>• Clearly specifies the step-by-step actions. *<br>• Suggests providing one final opportunity for researchers for professionally editing the comment as suggested by the reviewer, and if the researcher fails to abide by this, the journal should consider the case closed and informing the parties concerned. *<br>• Suggests pausing any decision on issuing retraction/notice until the institutional investigation is completed.	• Suggests adding a comment to the peer reviewer as follows: "Based on your review, if the Comment is revised to focus solely on science/methods, would its publication, with a Reply, be of net scholarly value to readers, or would it primarily serve a contentious, non-scholarly purpose?". *<br>• Suggests providing one final opportunity for researchers for professionally editing the comment as suggested by the reviewer, and if the researcher fails to abide by this, the journal should consider the case closed and informing the parties concerned. *<br>• Did not state that in case the journal wishes to follow up on the veto power of the researcher, they must ask for appropriate proof (stated in the COPE forum response).
	Stochastic	• Agrees that the journal has taken appropriate action.<br>• Clearly specifies the step-by-step actions. *	• Suggests providing one final opportunity for researchers for professionally editing the comment as suggested by the reviewer, and if the researcher fails to abide by this, the journal

		• States that veto right is part of the institutional/collaboration agreement and not publication ethics. *<br>• Suggests pausing any decision on issuing retraction/notice until the institutional investigation is completed.<br>• Specifies that veto right is institutional/collaboration agreement and not publication ethics. *	should consider the case closed and informing the parties concerned. *<br>• Did not state that in case the journal wishes to follow up on the veto power of the researcher, they must ask for appropriate proof (stated in the COPE forum response).

*-Not stated in the COPE response; AOR-Author on record; ICMJE: International Council of Medical Journal Editors; and CRediT: Contributor Role Taxonomy.
